# Potential of Pumpkin Pulp Carotenoid Extract in the Prevention of Doxorubicin-Induced Cardiotoxicity

**DOI:** 10.3390/pharmaceutics17080977

**Published:** 2025-07-28

**Authors:** Milana Bosanac, Alena Stupar, Biljana Cvetković, Dejan Miljković, Milenko Čanković, Bojana Andrejić Višnjić

**Affiliations:** 1 Department of Histology and Embryology, Faculty of Medicine, University of Novi Sad, 21000 Novi Sad, Serbia; dejan.miljkovic@mf.uns.ac.rs (D.M.); bojana.andrejic-visnjic@mf.uns.ac.rs (B.A.V.); 2 Institute of Food Technology, University of Novi Sad, 21000 Novi Sad, Serbia; alena.tomsik@fins.uns.ac.rs (A.S.); biljana.cvetkovic@fins.uns.ac.rs (B.C.); 3 Institute for Pulmonary Diseases of Vojvodina, Put Doktora Goldmana 4, 21208 Sremska Kamenica, Serbia; 4 Department of Internal Medicine, Faculty of Medicine, University of Novi Sad, 21000 Novi Sad, Serbia; milenko.cankovic@mf.uns.ac.rs; 5 Institute for Cardiovascular Diseases of Vojvodina, Put Doktora Goldmana 4, 21208 Sremska Kamenica, Serbia

**Keywords:** animal models, doxorubicin, cardiotoxicity, heart, carotenoids, pumpkin, cucurbita, NTproBNP, troponin I

## Abstract

**Background/Objectives**: Doxorubicin is a chemotherapeutic agent whose clinical use is limited by side effects (SEs). The most common SE is doxorubicin-induced cardiotoxicity (DIC), for which there is still no prevention. The hypothesis arises that active substances of natural origin could influence DIC prevention by affecting several pathways of DIC occurrence. **Methods**: Thirty Wistar rats were divided into six groups (control, NADES (C8:C10) solvent, pumpkin pulp extract, doxorubicin, NADES (C8:C10) solvent–doxorubicin, and pumpkin pulp extract–doxorubicin). During the experiment, parameters of general condition, body, and heart weight were observed. Heart function parameters were monitored by measuring the levels of serum NT-pro-BNP, CK-MB, and hsTnT. Tissue damage was evaluated by determining the doxorubicin damage score and the expression of anti-cardiac troponin I, anti-Nrf2, anti-Bcl-2, anti-caspase-3, anti-COX2, and anti-Ki67 antibodies. **Results**: Doxorubicin administration led to impaired general condition of animals and increased the levels of NT-proBNP, CK-MB, hsTnT, and myocardium tissue damage of medium grade. Its administration induced apoptosis (as evidenced by elevated Casp3), reduced antiapoptotic Bcl-2 and troponin I expression in cardiomyocytes. Reduced Nrf2 expression due to doxorubicin administration was restored when pumpkin pulp extract containing carotenoids was coadministered, which led to the normalization of Casp3, Bcl-2, and troponin I expression. Consequently, the general condition and body weight were better in animals treated with both doxorubicin and the other treatment compared to those treated with doxorubicin alone. **Conclusions**: The results of this study strongly suggest that pumpkin pulp extract containing carotenoids has a cardioprotective effect, possibly by regulating the Nrf2 pathway.

## 1. Introduction

Doxorubicin (DOX) is a powerful chemotherapeutic agent from the anthracycline antibiotic group, whose clinical use is limited by the occurrence of acute and chronic side effects. The most common and severe chronic side effect is doxorubicin-induced cardiotoxicity (DIC). The incidence of DIC is dose-dependent, and its clinical significance increases with the increase in the incidence of cancer, longer survival, and the increasing use of doxorubicin in groups predisposed to the occurrence of cardiac dysfunction of a certain degree [1,2].

The mechanisms of DIC occurrence have not yet been completely elucidated, but there are two patterns that have been most extensively investigated [3]. Firstly, doxorubicin and its metabolites create reactive oxygen species (ROS), leading to oxidative stress (OS) when the presence of ROS prevails over the antioxidant mechanisms. The ROS increase is particularly significant and harmful in cardiomyocytes, where the levels of antioxidant protection are low [4,5]. Damage at the mitochondrial level occurs similarly, due to excessive accumulation of ROS, either through disruption of the mitochondrial membrane system or direct impact on the complexes within the mitochondrial electron transport system (ETS) [6,7,8]. Given that DOX preferentially accumulates in mitochondria, its high numbers, combined with less active antioxidant defenses in the heart, potentially explain why DIC is the most common adverse effect of doxorubicin [8]. Secondly, doxorubicin damages topoisomerase II, thus forming unwanted double-strand breaks that have a toxic effect on cells, consequently leading to apoptosis [9]. More recent research on DIC emphasizes the role of regulated cell death (RCD), such as apoptosis, necrosis, autophagy, and ferroptosis.

Research has focused on testing different medications and supplements, but little progress has been made in preventing DIC so far. This suggests that the investigated substances affect only one of the mechanisms of DIC, and perhaps not to a full extent. Therefore, it is vital that new medications and natural products become the topic of preclinical research on DIC prevention, and carotenoids have been singled out in this sense in modern research. Carotenoids are fat-soluble pigments produced by plants, algae, and several bacteria and fungi [10]. When used in animal model studies, several facts should be taken into consideration. The processes of extraction and solubilization are critical for the efficient absorption of dietary carotenoids and often determine the overall bioavailability of these nutrients [11,12]. Therefore, it is of paramount significance to optimize extraction and seek new solvents for extracted carotenoids.

Pumpkin (*Cucurbita moschata*) is a readily available plant material that can be used to obtain a high-value extract rich in carotenoids [13], but the challenge lies in preserving the carotenoids. For example, β-carotene, the most abundant carotenoid in pumpkin, is poorly soluble in water, sensitive to oxygen, heat, and light. Therefore, it is essential to find optimal extraction methods and conditions to achieve maximum concentrations of bioactive compounds and preserve their bioactivity for further application [14]. Regarding the bioavailability of carotenoids in the body, the key factor is the matrix, i.e., the environment in which the carotenoids are located, which, in the case of extracts, is the solvent.

The traditional, so far most commonly used methods of carotenoid extraction often require large amounts of non-selective, toxic, and environmentally unacceptable organic solvents [14]. The latest generation of “green” solvents—natural deep eutectic solvents (NADES)—are currently in the special focus of research because only natural, nontoxic, biodegradable compounds are used for the formation of NADES solvents [15], the preparation is simple, and there is the possibility of adjusting the properties of the solvent to achieve efficient extraction and stabilization of natural products [14]. These characteristics of NADES make them promising solvents for the extraction of carotenoids.

Absorption also varies among species, and while gerbils, ferrets, and preruminant calves are the best models for studying carotenoid absorption, these models are not well established for the study of diseases with epidemiologic correlations to carotenoids. Models using mice and/or rats are more established for studying cancer, immune function, and vitamin A deficiency. In humans, roughly 20–75% of the β-carotene is absorbed intact, but most laboratory animals break down β-carotene in their intestines and thus absorb almost none intact [16]. Once absorbed, carotenoids undergo enzymatic cleavage by carotenoid cleavage oxygenase (CCO) family members, which are present in all kingdoms of life [10]. A conversion of provitamin A carotenoids into retinal, a form of vitamin A, is carried out by β-carotene-15,15′-dioxygenase (BCO1), while another enzyme, β-carotene-9′,10′-oxygenase (BCO2), has a much broader spectrum of substrates, including both provitamin A and non-provitamin A carotenoids. Both enzymes are primarily present in the intestines. While BCO1 is cytoplasmic, BCO2 is placed in the inner mitochondrial membrane [17]. So far, over 1100 different carotenoids have been discovered, not counting so-called apo-carotenoids (carotenoid derivatives and breakdown products generated through the action of CCO) [18]. There are opinions that apocarotenoids, too, could induce biological effects in humans and rodents [17,19].

Studies on DIC prevention should aim to identify such molecules that can affect several aspects of DIC prevention. Recent research suggests that carotenoids or compounds derived from them after the action of CCO enzymes could be such a type of molecule. Firstly, they have an antioxidant capacity [20,21]. Secondly, studies show that pumpkin carotenoids reduce changes in various mitochondrial genes, protecting the integrity of the ETC and maintaining cellular viability [22]. This is believed to be achieved both by direct neutralization of ROS and by regulating the nuclear factor erythroid 2-related factor 2/heme oxygenase 1 (Nrf2/HO1) pathway. Nrf2 is a transcription factor responsible for regulating the cellular redox balance, and its activation is a key component of the oxidative stress response cascade signaling [23]. Doxorubicin in mice induces ferroptosis by activating the Nrf2 pathway, leading to an increase in heme oxygenase 1 (HO-1). Heme oxygenase catalyzes the degradation of heme, resulting in the release of free iron and the accumulation of oxidized lipids within the mitochondrial membrane. Conflicting studies indicate that dysfunction in the Nrf2 pathway is closely related to DIC, concluding that the Nrf2/HO1 signaling pathway can be a potential drug target for the prevention and/or treatment of DIC [24]. Finally, DOX-induced ROS overproduction activates mitochondrial regulation of apoptosis, while carotenoids, such as lycopene and β-carotene (and/or their derivatives), prevent the loss of antiapoptotic proteins (Bcl-2, Bcl-xL) [16].

From all the above, the hypothesis arises that pumpkin pulp extract containing carotenoids could provide multifactorial prevention of DIC by regulating Nrf2, reducing OS, and preventing mechanisms of regulated cell death.

## 2. Materials and Methods

### 2.1. Extraction Procedure

A natural deep eutectic solvent (NADES) was used for the extraction, prepared by combining octanoic and decanoic acids in a 3:1 ratio (C8:C10). This solvent was selected based on its proven optimal affinity and solubility for β-carotene, as reported in the literature [18,19,20]. The components of the NADES solvent were carefully mixed in the specified ratio and gently heated to 50 °C, with continuous stirring. The mixture was allowed to homogenize completely, after which the NADES solvent was slowly cooled to room temperature.

To enhance the extraction efficiency and increase the β-carotene yield from pumpkin, a combination of NADES solvent and ultrasound-assisted extraction (UAE) was employed. The UAE process was carried out using an ultrasonic water bath (Elmasonic P30, Elma Hans Schmidbauer GmbH & Co. KG, Singen, Germany) operating at a fixed frequency of 37 kHz. Extraction parameters were optimized according to the procedure outlined by Stupar et al. [21], utilizing a response surface methodology at five levels to determine the optimal conditions. For maximum carotenoid yield, the following extraction parameters were applied: extraction temperature of 50 °C, ultrasound power of 52.5 W/cm^3^, extraction time of 10 min, and a NADES solvent to matrix ratio of 7 mL/g.

### 2.2. Total Carotenoid Content

The content of total carotenoids in the obtained extract was determined by the spectrophotometric method [22]. β-carotene was used to construct the calibration curve, while the total carotenoid content was expressed as β-carotene equivalent (μg β-carotene/g pumpkin powder).

### 2.3. Determination of Carotenoid Profiles Using High-Performance Liquid Chromatography (HPLC)

The composition and quantification of carotenoids in the extract obtained under optimal conditions were analyzed according to previously described methods [23]. An Agilent 1200 series HPLC system (Agilent Technologies, Santa Clara, CA, USA) equipped with a diode array detector and a Zorbax SB C18 column (3.0 × 250 mm, particle size = 5 μm) was used. Separation of carotenoids was performed at a temperature of 24 ± 1 °C, with a flow rate of 1.5 mL/min. Two eluents were used: A—acetone/water (75:25, *v*/*v*) and B—acetone/methanol (75:25, *v*/*v*), with a gradient profile: from 0 to 25% B, 10 min; from 25 to 100% B, 35 min; and 100% B, 10 min. The method used was validated using carotenoid standards in terms of linearity, limit of detection, limit of quantitation, precision, and accuracy. Peaks were identified by comparing their choline retention times and spectra with carotenoid standards and literature data.

### 2.4. Experimental Animals and Ethical Statement

For the experiment, healthy adult male white laboratory rats of the Wistar strain were used. At the beginning of the experiment, the rats weighed over 300 g. The animals were obtained from the Military Medical Academy (Belgrade, Serbia) and housed in Ehret Uni-Protect cabinets equipped with a high-efficiency particulate air (HEPA) filter system (EHRET Labor- und Pharmatechnik GmbH & Co. KG, Emmendingen, Germany). The animals were housed in polycarbonate transparent cages with a standard circadian cycle (12 h day–12 h night), controlled temperature (22–25 °C), and relative humidity (55 ± 1.5%), and had free access to pelleted food and water.

All experimental procedures and animal care were conducted following the ethical principles outlined in the EU Directive 2010/63/EU on animal welfare and in compliance with the Law on Animal Welfare of the Republic of Serbia (OG RS 41/09). The Ethics Committee approved the study design involving animals for the protection of the welfare of laboratory animals at the University of Novi Sad (Novi Sad, Serbia), after which the Ministry of Agriculture, Forestry, and Water Management of the Republic of Serbia issued Decision No. 323-07-10388/2021-05 on the approval of the implementation of the planned experiment on animals.

### 2.5. In Vivo Experimental Design

The experiment was performed on 30 rats, randomly divided into 6 groups:

1. Negative control group (C): they received daily, once a day, per os (orogastric tube), 1 mL of saline from the 1st to the 20th day of the experiment (0.9% NaCl);
2. NADES solvent (C8:C10) (N): they received daily, once a day, per os (orogastric tube), 1 mL of NADES solvent from the 1st to the 20th day of the experiment;
3. Pumpkin pulp extract (P): they received daily, once a day, per os (orogastric tube), 900 µg/kg of pumpkin pulp extract from the 1st to the 20th day of the experiment;
4. Positive control group—doxorubicin (D): they received daily, once a day, per os (orogastric tube), 1 mL of saline from the 1st to the 20th day of the experiment (0.9% NaCl), as well as 4 doses of doxorubicin (2 mg/kg, intraperitoneally) on the 8th, 12th, 16th, and 20th day of the experiment;
5. NADES solvent (C8:C10) and doxorubicin (ND): they received daily, once a day, per os (orogastric tube), 1 mL of NADES solvent from the 1st to the 20th day of the experiment, as well as 4 doses of doxorubicin (2 mg/kg, intraperitoneally) on the 8th, 12th, 16th, and 20th day of the experiment;
6. Pumpkin pulp extract and doxorubicin (PD): they received daily, once a day, per os (orogastric tube), 900 µg/kg of pumpkin pulp extract from the 1st to the 20th day of the experiment, as well as 4 doses of doxorubicin (2 mg/kg, intraperitoneally) on the 8th, 12th, 16th, and 20th day of the experiment. On days when both doxorubicin and the extract were applied, the interval between the two substances was 4 h.

Doses of test substances administered to animals were converted to equivalent human therapeutic doses using the Food and Drug Administration (FDA)-approved human–animal dose conversion formula, assuming a human body weight of 70 kg [24].

### 2.6. General Condition, Body Weight, Heart Weight, and Sacrifice of Animals

To monitor the mental and physical health of the animals during the experiment, the following observations were made: the quality and density of the hair, the presence of bleeding, and the animals’ mood. The amount of blood obtained by exsanguination was also compared, as well as the amount and properties of serum. Animals were anesthetized on the 21st day with urethane (0.75 g/kg, intraperitoneally). After induction of anesthesia, cardiopuncture and sacrifice via exsanguination were performed (21st day). The body weight (BW) of the animals was measured at the beginning (1st day) of the experiment and on the day of sacrifice (24 h after the last administered treatment dose, on the 21st day of the experiment). Also, the heart weight (HW) was measured on the 21st day of the experiment.

### 2.7. Examination of Biochemical Markers

In order to investigate the toxic effect of doxorubicin on the heart, biochemical markers of cardiac damage—N-terminal pro b-type natriuretic peptide (NT-proBNP), creatine kinase-MB (CK-MB), and high-sensitivity cardiac troponin (hsTns)—were also measured. All analyses were performed according to standard spectrophotometric methods on an automatic system for chemical analysis, following the instructions provided by the manufacturers of the respective commercial kits.

### 2.8. Histological Processing of Tissues and Immunohistochemical Staining of Preparations

After sampling, the heart tissue samples were fixed in 4% buffered formalin for 24 h. Subsequently, the process of dehydration with isopropyl alcohol of different concentrations (70%, 80%, 96%, and absolute) was initiated. After that, the tissue was placed in paraffin at a temperature of 56 °C to permeate the tissue overnight. On the fifth day, the tissue was cast in paraffin blocks on a casting console (Sakura Finetek, Chuo-ku, Tokyo, Japan). The tissue was then cut into 5 µm slices on a microtome (Sakura Finetek, Chuo-ku, Tokyo, Japan), transferred to a water bath (DiaPath, Martinengo, Italy), after which the slices were transferred to a glass slide and a thermostat (Memmert GmbH Co. KG, Büchenbach, Germany), and then stained with standard histological staining—hematoxylin and eosin (H&E).

In addition to standard (H&E) staining, immunohistochemical staining using primary antibodies was applied to tissue samples: anti-cardiac troponin I (TnI) (ab209809); anti-Nrf2 (Nrf2) (ab31163); anti-Bcl-2 (Bcl2) (ab196495); anti-caspase-3 (Casp3) (ab179517); anti-COX2 (COX2) (ab283574); anti-Ki67 (Ki67) (ab279653), Abcam, Cambridge, UK.

### 2.9. Semiquantitative Analysis of H&E Preparations

**Figure 1:**
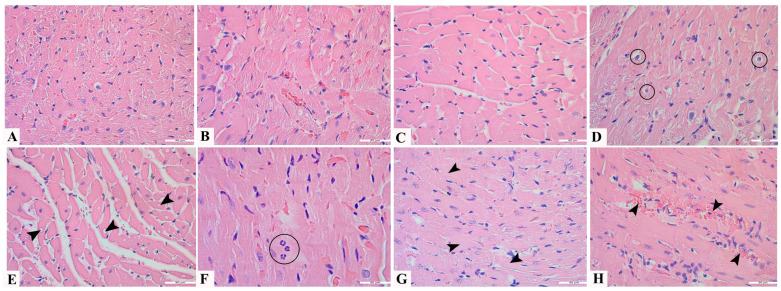
Pathohistological indicators of myocardial damage after doxorubicin treatment: (**A**) disorganization of cardiomyocytes (all cells), 400×; (**B**) disorganization of myofilaments (all cells), 400×; (**C**) presence of intestinal edema and edema of cardiomyocytes (all cells), 400×; (**D**) changes in the appearance of nuclei (circle), 400×; (**E**) vacuolization (arrowhead), 400×; (**F**) infiltration of the myocardium by neutrophils (circle), 630×; (**G**) necrosis (arrowhead), 400×; (**H**) hemorrhage (arrowhead), 400×.

The following changes in the myocardium of the heart were analyzed (Figure 1) on H&E stained tissue slides: disorganization of cardiomyocytes (Figure 1A) and myofilaments (Figure 1B), presence of intestinal edema and edema of cardiomyocytes (Figure 1C), changes in the appearance of nuclei (Figure 1D), vacuolization (Figure 1E), infiltration of the myocardium by neutrophils (Figure 1F), necrosis (Figure 1G), and hemorrhage (Figure 1H). Scoring of observed changes, calculation, and interpretation of the final doxorubicin damage score (DDS) used to measure myocardial tissue damage, was performed according to the methodology established by Bosanac and associates [25].

Based on the score value, it is interpreted as follows:

- negative DDS score (score < 2), i.e., absence of myocardial damage caused by doxorubicin, or
- positive DDS score (score 2–7), existence of damage caused by doxorubicin, namely:
  1. mild damage (score 2–3.99),
  2. moderate damage (score 4–5.99), or
  3. high grade damage of a strong/pronounced degree (score 6–7).

### 2.10. Semiquantitative Analysis of Immunohistochemically Stained Preparations

For analysis on immunohistochemically stained preparations, a semiquantitative evaluation of 3 expression parameters was performed:

- maximum staining intensity (Imax): the strongest staining intensity among all stained cells—mild (1), moderate (2), and strong (3);
- dominant staining intensity (Idom): staining intensity observed in the largest number of stained cells—mild (1), moderate (2), and strong (3);
- staining extent (Iext): percentage of cells showing positive staining—<25% of cells are positive (1), 25–50% of cells are positive (2), 50–75% of cells are positive (3), and >75% of cells are positive (4).

The final score for each tissue section was obtained as the sum of the scores for each parameter.

### 2.11. Statistical Processing and Analysis of the Obtained Results

Among the methods for testing statistical hypotheses, the parametric method of analysis of variance ANOVA was used to prove the difference between three or more groups. As non-parametric statistical methods, the Mann–Whitney test was used to determine the difference between two groups, the Kruskal–Wallis test to determine the difference between three or more groups, and the Wilcoxon test for matched pairs. Hypotheses were tested at the level of statistical significance (*p* level) of *p* < 0.01, *p* < 0.001, and *p* < 0.05. The results are presented in tables and figures. Data were processed using standard statistical packages (IBM SPSS Statistics 26).

## 3. Results

### 3.1. Qualitative and Quantitative Analysis

Qualitative and quantitative analysis of the chemical composition of the extract have demonstrated its potential as a promising alternative to conventional organic solvents for carotenoid extraction. The application of solid–liquid extraction, such as maceration, using NADES (C8:C10) solvents resulted in significantly higher carotenoid content compared to traditional solvents, such as hexane. Additionally, the intensification of extraction with a combination of NADES (C8:C10) and ultrasonic extraction resulted in a further improvement in the extraction yield. The characterization of the obtained extract included spectrophotometric analysis, which showed a total carotenoid content of 346.4616 μg/mL. Further HPLC analysis determined the profile of carotenoids, with α-carotene and β-carotene being the dominant compounds, accounting for more than 90% of the total detected carotenoids. The content of carotenoids detected by the HPLC method is β-carotene: 114.54 μg/mL and α-carotene: 147.91 μg/mL (expressed as beta equivalent: μg β-carotene/mL). Given their significant presence, these two carotenoids were the focus of further research.

### 3.2. General Condition of Animals

**Figure 2:**
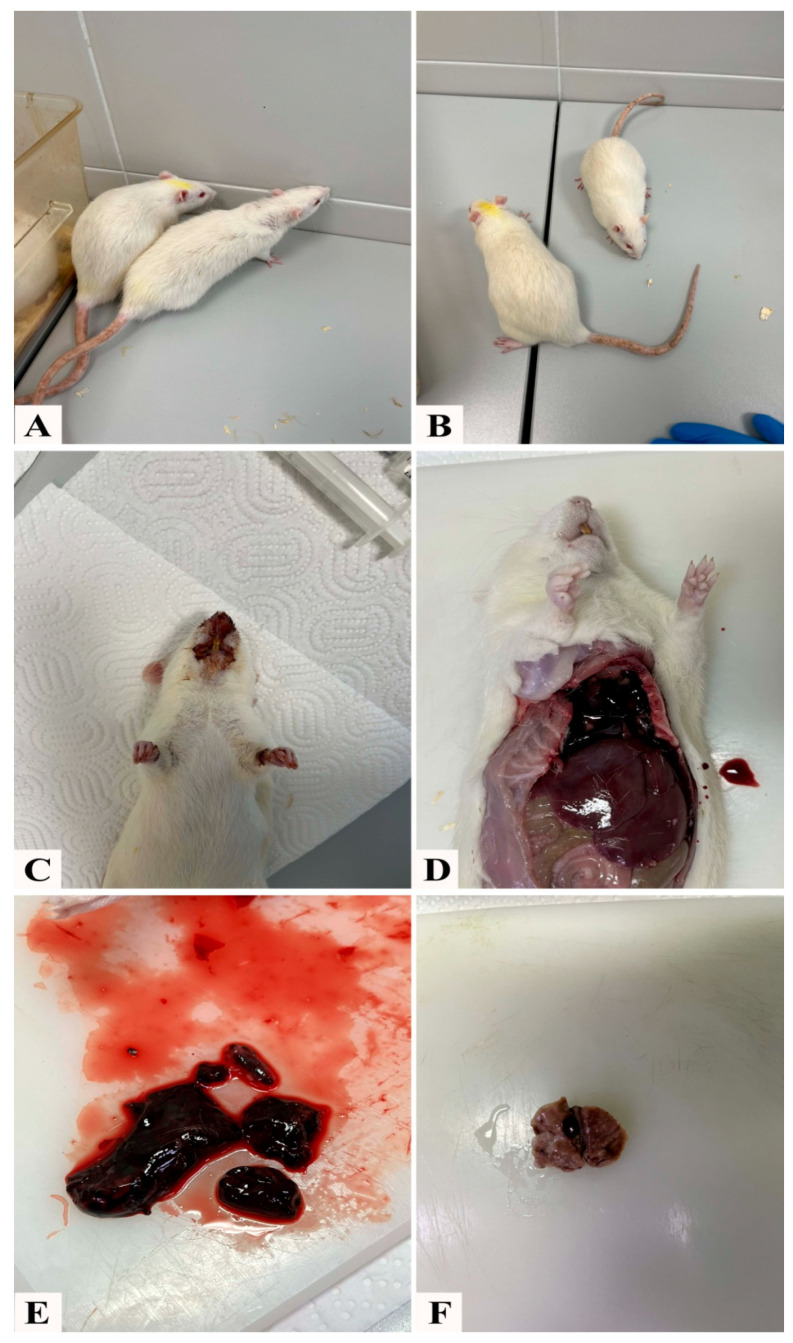
(**A**) Animals from the D group; (**B**) animals from the PD group; (**C**) D group (traces of bleeding from the nose); (**D**) D group (autopsy; the chest and abdominal cavity); (**E**) D group (pleural content); (**F**) D group, connective tissue, pleural adhesions.

All animals in the control group, treated only with saline, remained healthy throughout the experiment, and no changes in their general condition were observed. In animals of group D, after administration of toxic doses of doxorubicin, a significant deterioration of the general condition was observed, which was reflected in the following: thinned fur, of significantly worse quality compared to group PD (Figure 2A,B), traces of bleeding from the nose (dried blood on the muzzle) (Figure 2C), and gastrointestinal tract (blood in the stool). The animals were lethargic and did not show signs of fighting for life. During the necropsy of group D animals, necrosis was observed at the injection sites of doxorubicin, along with the presence of serohemorrhagic content and coagulum in the chest and massive adhesions (Figure 2D–F). The presence of ascites in the abdomen of group D animals is believed to be the reason for their poor response to anesthesia. The amount of blood obtained by exsanguination, as well as the amount of serum obtained after centrifugation, was notably lower in group D. In group D, 1–1.5 mL of blood was obtained, while in animals of other groups, more than 2.5 mL was obtained. The application of the NADES solvent without doxorubicin (group N) did not affect the general condition of the animals, and all animals remained healthy, similar to those in the control group, indicating that its use is safe and nontoxic. The serum of animals of both group D and ND was more hemolyzed compared to the control, N, P, and PD groups. Treatment with the extract of pumpkin pulp strongly neutralized the toxic effect of doxorubicin, and in the PD group, no observed changes were noted in any animal. Likewise, the extract contributed to a better quality of fur in animals of the P group and did not negatively affect the general condition of the animals.

### 3.3. Body Weight (BW) and Heart Weight (HW)

The values of the body and heart weight of animals at the beginning and end of the experiment, as well as the differences in body weight, are shown in Table 1.

**Table 1:** Average body and heart weight of animals at the beginning of the experiment (day 1) and immediately before sacrifice (day 21) ($\bar{X}$ ± SD).

Group	BW (1-Day) (g)	BW (21-Day) (g)	∆BW	HW (g)
C	429.00 ± 31.25	475.88 ± 30.43	46.88	1.48 ± 0.21
N	301.38 ± 25.12	380.25 ± 51.63	78.87	1.56 ± 0.19 ^#^
P	301.13 ± 23.87	339.13 ± 43.69	38	1.23 ± 0.18 *
D	401.13 ± 37.84	419.50 ± 32.47	18.37 *	1.32 ± 0.16
ND	320.88 ± 38.45	334.38 ± 40.71	13.5	1.05 ± 0.20 *^,#^
PD	304.00 ± 26.15	317.50 ± 50.32	13.5	1.64 ± 1.35

* statistically significant difference compared to group C, *p* < 0.05. ^#^ statistically significant difference compared to group D, *p* < 0.05.

In all groups, the average body weight increased, with statistically significant differences in body weight gain (∆BW). Animals treated with toxic doses of doxorubicin had a statistically significantly lower body weight gain compared to the control group (*p* < 0.05). Co-treatment with pumpkin pulp extract led to a greater BW gain in the PD group compared to the D group, but these differences were not statistically significant. Animals of the C, N, and P groups at the end of the experiment had a higher BW gain than at the beginning of the experiment, with the highest values observed in the N group (statistically significantly higher compared to the C and P groups) (*p* < 0.05).

The heart weight of animals treated with doxorubicin was lower compared to the control group and statistically significantly lower compared to the N group (*p* < 0.05). This difference did not affect the general condition of the animals, which was similar to that of the control group. Administration of doxorubicin with the NADES solvent leads to a statistically significant decrease in heart weight compared to administration of solvent alone (N group). It seems surprising that the heart weight of animals treated only with the extract had a statistically significantly lower heart weight compared to the control group. However, these data should be taken into account because the weight of the animals in this group is also less than that of the animals in the control group, so it may be a result of purely anatomical variations. The heart weight of the animals that received pumpkin extract along with doxorubicin did not differ statistically from the heart weight of the control animals or the animals in group D.

### 3.4. Assessment of Cardiovascular Biomarkers (NT-proBNP, CK-MB, hsTnT)

**Figure 3:**
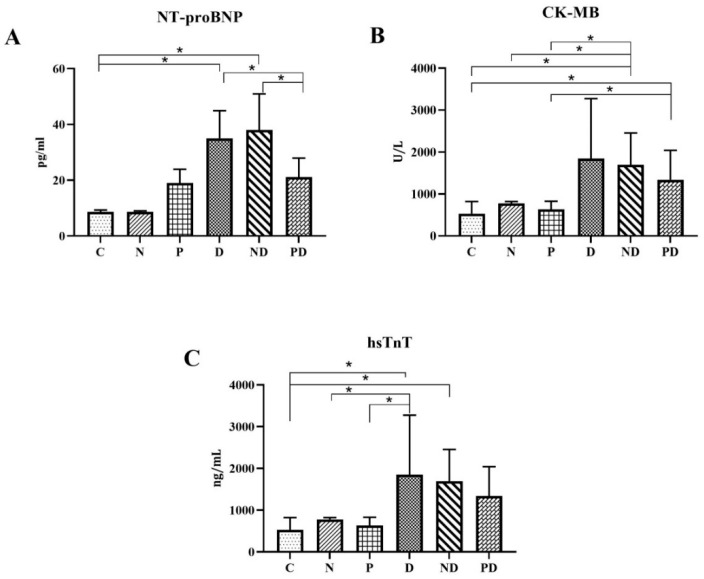
(**A**) Serum NT-proBNP (pg/mL ± SD); (**B**) serum CK-MB (U/L ± SD); and (**C**) serum hsTnT (ng/mL ± SD) values in heart tissue of rats treated with saline (C), NADES solvent (N), pumpkin pulp extract (P), doxorubicin (D), NADES solvent and doxorubicin (ND), and pumpkin pulp extract and doxorubicin (PD). * *p* < 0.001.

Application of four toxic doses of doxorubicin in groups D and ND led to statistically significantly higher values of NT-proBNP levels compared to groups C, N, and P. In the PD group, co-treatment with pumpkin pulp extract statistically significantly reduced the high levels of NT-proBNP caused by doxorubicin, compared to the D and ND groups (*p* < 0.001, Figure 3A).

CK-MB values were notably higher in all groups where doxorubicin was administered, compared to the C, N, and P groups. Although the average value of CK-MB was the highest in the D group, compared to the control, statistical significance is absent, which may be due to the variation among individual values in the group, as indicated by the significant standard deviation. The PD group, which received doxorubicin and the extract, had a lower average value of CK-MB compared to the D group, but still statistically significantly higher values compared to the control (*p* < 0.001, Figure 3B).

Treatment of animals with toxic doses of doxorubicin (D group) led to statistically significantly higher hsTnT values compared to C, N, and P groups (*p* < 0.001). Co-treatment with pumpkin pulp extract and doxorubicin (group PD) led to a decrease in hsTnT levels compared to groups D and ND. However, these differences were not statistically significant (Figure 3C).

By analyzing the values of CK-MB, NT-proBNP, and hsTnT, it was observed that NADES alone does not cause an increase in values, which indicates that this natural solvent does not lead to disturbances in cardiovascular biomarkers, i.e., it does not have cardiotoxic properties, and that the increased values of biomarkers in the ND group are the result of the action of doxorubicin, the action of which NADES does not mitigate or potentiate.

### 3.5. Doxorubicin Damage Score (DDS)

**Figure 4:**
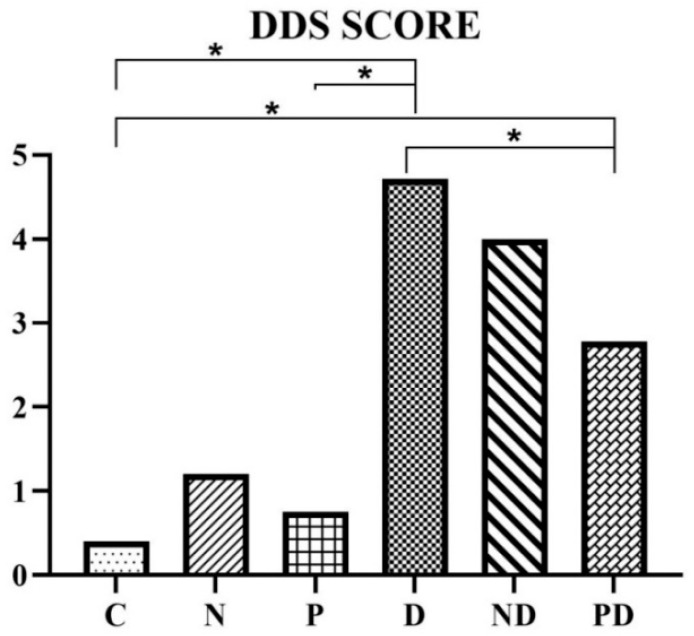
Average values of DDS score in heart tissue of rats treated with saline (C), NADES solvent (N), pumpkin pulp extract (P), doxorubicin (D), NADES solvent and doxorubicin (ND), and pumpkin pulp extract and doxorubicin (PD). * *p* < 0.001.

Taking into account all analyzed parameters in each experimental animal, the DDS score was calculated, showing the average level of myocardial damage for each group (Figure 4). As expected, the lowest average DDS score (<2, i.e., negative DDS) was observed in groups C and P, and the highest in group D (4–5.99, positive DDS, i.e., moderate damage), which was found to be a statistically significant difference in the level of myocardial damage. Group ND, with an average score of 4.0, corresponds to a moderate degree of damage in this group, which falls within the range and is comparable to the D group. It is statistically significantly higher compared to the control group, as well as to groups N and P (*p* < 0.001). The application of pumpkin pulp extract managed to statistically significantly lower the DDS score (2.78), and the changes in the myocardium in the PD group correspond to mild damage (DDS score 2–3), which is statistically significantly lower compared to the D group, but still statistically significantly higher compared to the C, N, and P groups (*p* < 0.001).

By analyzing the DDS score values, it was observed that NADES alone does not cause an increase, which indicates that this natural solvent does not lead to myocardial damage, i.e., it does not exhibit cardiotoxic properties. The increased values in the ND group are the result of the action of doxorubicin, which is neither mitigated nor potentiated by NADES.

### 3.6. Immunohistochemistry

#### 3.6.1. Anti-Cardiac Troponin I (TnI)

**Figure 5:**
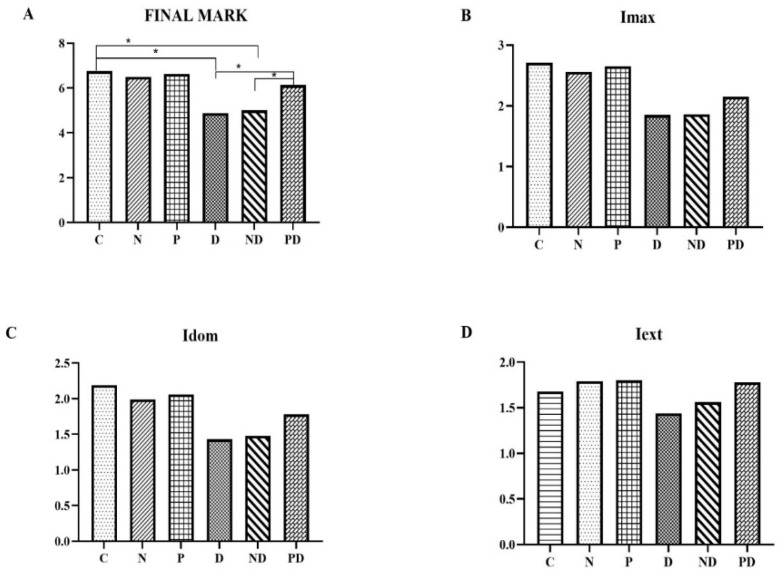
Overall score and results of individual parameters of anti-cardiac troponin I expression in heart tissue of rats treated with saline (C), NADES solvent (N), pumpkin pulp extract (P), doxorubicin (D), NADES solvent and doxorubicin (ND), and pumpkin pulp extract and doxorubicin (PD): (**A**) Final mark; (**B**) Imax—maximum staining intensity; (**C**) Idom—dominant staining intensity; (**D**) Iext—staining extent.* *p* < 0.001.

**Figure 6:**
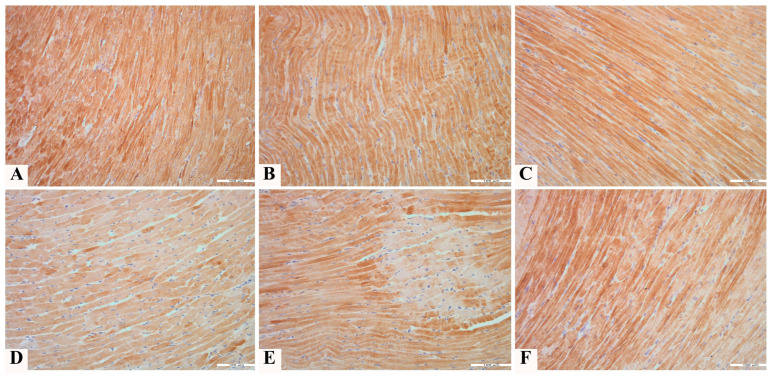
Anti-cardiac troponin I expression in heart tissue of rats treated with (**A**) saline (C); (**B**) NADES solvent (N); (**C**) pumpkin pulp extract (P); (**D**) doxorubicin (D); (**E**) NADES solvent and doxorubicin (ND); and (**F**) pumpkin pulp extract and doxorubicin (PD), 200×.

No statistically significant differences in the expression of TnI were observed between the animals treated with the NADES solvent and the extract from pumpkin pulp, compared to the control group. The application of toxic doses of doxorubicin (groups D and ND) resulted in a statistically significant decrease in the expression of this antibody compared to groups C, N, and P (*p* < 0.001). Co-treatment with pumpkin pulp extract (PD group) reduced the toxic effect of doxorubicin. Although a statistically significant increase in expression of troponin I was observed in the PD group compared to group D, the expression level of the PD group remained significantly lower compared to groups C, N, and P (*p* < 0.001). Regarding the expression of TnI, pumpkin pulp extract had the most effect on preserving the expression in a larger number of cells, that is, staining extensiveness (Iext) (Figure 5 and Figure 6).

#### 3.6.2. Anti-Nrf2 (Nrf2)

**Figure 7:**
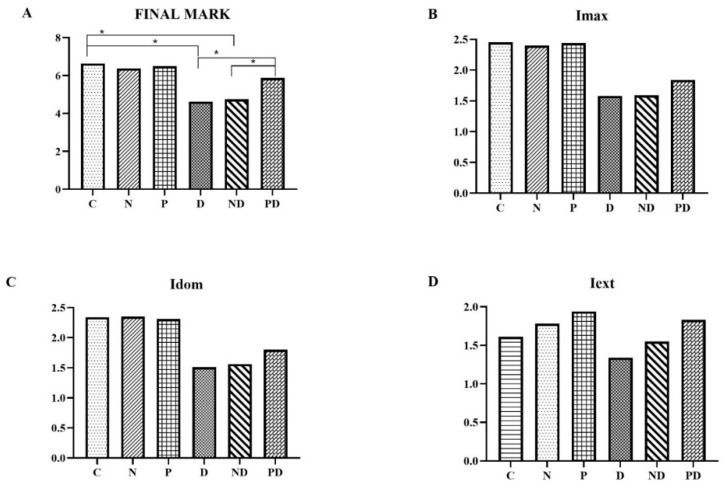
Overall score and results of individual parameters of anti-Nrf2 expression in heart tissue of rats treated with saline (C), NADES solvent (N), pumpkin pulp extract (P), doxorubicin (D), NADES solvent and doxorubicin (ND), and pumpkin pulp extract and doxorubicin (PD): (**A**) Final mark; (**B**) Imax—maximum staining intensity; (**C**) Idom—dominant staining intensity; (**D**) Iext—staining extent. * *p* < 0.001.

**Figure 8:**
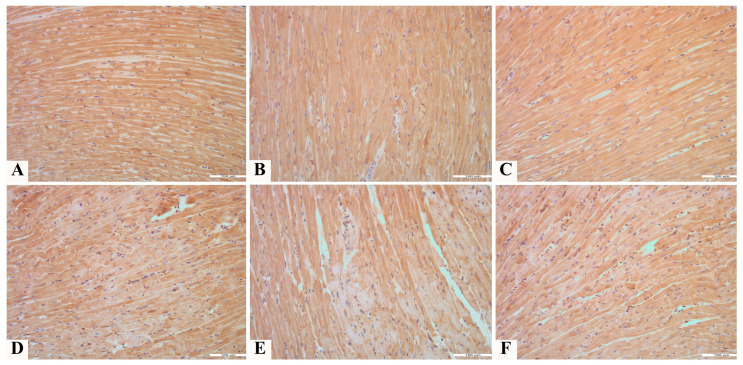
Anti-Nrf2 expression in heart tissue of rats treated with (**A**) saline (C); (**B**) NADES solvent (N); (**C**) pumpkin pulp extract (P); (**D**) doxorubicin (D); (**E**) NADES solvent and doxorubicin (ND); and (**F**) pumpkin pulp extract and doxorubicin (PD), 200×.

The expression of Nrf2 did not statistically differ among the C, N, and P groups. Treatment with toxic doses of doxorubicin in the D and ND groups led to a statistically significant decrease in the expression of Nrf2 in the cytoplasm of cardiomyocytes compared to groups C, N, and P (*p* < 0.001). The co-treatment with pumpkin pulp extract and doxorubicin resulted in a statistically significant increase in Nrf2 expression compared to groups D and ND (*p* < 0.001). Nevertheless, the Nrf2 expression in the PD group remained statistically significantly lower than in groups C, N, and P (*p* < 0.001). As with the application of TnI, the application of the extract had the greatest effect on preserving the expression of Nrf2 in a larger number of cells, as indicated by the extent of staining (Iext) (Figure 7 and Figure 8).

#### 3.6.3. Anti-Bcl-2 (Bcl2)

**Figure 9:**
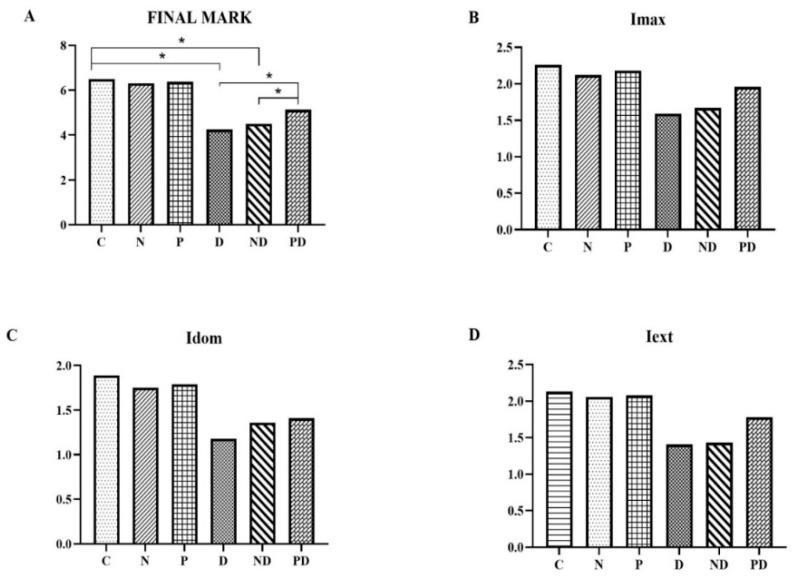
Overall score and results of individual parameters of anti-Bcl-2 expression in heart tissue of rats treated with saline (C), NADES solvent (N), pumpkin pulp carotenoid extract (P), doxorubicin (D), NADES solvent and doxorubicin (ND), and pumpkin pulp extract and doxorubicin (PD): (**A**) Final mark; (**B**) Imax—maximum staining intensity; (**C**) Idom—dominant staining intensity; (**D**) Iext—staining extent. * *p* < 0.001.

Administration of toxic doses of doxorubicin led to a statistically significant decrease in Bcl2 expression compared to groups C, N, and P (*p* < 0.001). Additionally, treatment with NADES solvent did not alter the toxic effect of doxorubicin; therefore, in groups ND and D, a statistically significant decrease in the expression of this antibody was observed compared to groups C, N, and P. The doxorubicin-induced decreased expression was statistically significantly increased by the use of pumpkin pulp extract compared to group D (*p* < 0.001). Of the observed parameters of Bcl2 expression, the application of pumpkin pulp extract contributed the most to the increase of the maximum staining intensity (Imax), as well as to the preservation of expression in a large number of cells (Iext) (Figure 9).

#### 3.6.4. Anti-Caspase-3 (Casp3), Anti-COX2 (COX2), and Anti-Ki67 (Ki67)

The ANOVA test showed a statistically significant difference in the average values of the number of Casp3-, COX2-, and Ki67-positive cells/mm^2^ in different groups of animals, while comparing the median expression intensity of Casp3 (Suplementary Figure S1), COX2 (Suplementary Figure S2), and Ki67 (Suplementary Figure S3) a statistically significant difference was found between the groups (Table 2).

Post hoc analysis using the Tukey HSD test revealed a statistically significant increase in the average number of Casp3-, COX2-, and Ki67-positive cells in animals treated with toxic doses of doxorubicin (groups D and ND) compared to C, N, and P (*p* < 0.001). On the other hand, the treatment with pumpkin pulp extract alleviated the toxic effects of doxorubicin. A statistically significant decrease in the number of Casp3-positive cells in the PD group compared to the D group was verified, as well as a decrease in the number of Ki67-positive cells compared to groups D and ND, and a decrease in COX2-positive cells was observed in the PD group compared to the ND group.

Further analysis revealed that toxic doses of doxorubicin (group D) influenced a statistically significant increase in the expression of Casp3, predominantly of moderate intensity, compared to groups C, N, P, and ND, as well as the expression of Ki67 and COX2 intensity compared to the C, N, and P groups. In the group of animals treated with pumpkin pulp extract and doxorubicin (the PD group), a statistically significant decrease in the expression of Casp 3 and COX2 was observed compared to the D group, as well as a decrease in Ki67 expression compared to the C, N, and P groups.

By analyzing the expression of TnI, Nrf2, Bcl2, Casp3, COX2, and Ki67, it was observed that NADES alone does not cause an decrease in expression of TnI, Nrf2, and Bcl2, as well as increase in expression of Casp3, COX2, and Ki67, which indicates that this natural solvent does not lead to myocardium damage and that the decreased or increased expression of different antibodies in the ND group are the result of the action of doxorubicin, which is neither mitigated nor potentiated by NADES.

## 4. Discussion

It is known that food rich in carotenoids has a preventive effect on various diseases [25]. Due to the high content of carotenoids, pumpkin occupies a special status in the domain of functional foods with potential medicinal value. However, consumption of foods rich in carotenoids, such as β-carotene, often results in low bioavailability of these compounds from plant sources (10–65%) due to the resistance of carotene–protein complexes, fibers, and plant cell walls to digestion and degradation [26,27]. In order to improve the bioavailability of carotenoids from plants, they can be used in the form of plant extracts. Pumpkin is an extremely affordable and rich source of carotenoids that allows the production of extracts with high carotenoid content [28].

This research indicated that the choice of extraction technique and solvent can improve the content of carotenoids, the stability of the extract, and its long-term quality, ensuring that the potential health benefits of carotenoids are maximized. The profile and concentrations of carotenoids differ depending on the type of pumpkin [29]. This research utilized butternut squash (*C. moschata*), and the intensification of extraction with a combination of NADES (C8:C10) and ultrasonic extraction was employed. The total carotenoid content was 346.4616 μg/mL in the extract. Further analysis revealed that the presence of β-carotene and α-carotene was in much higher concentrations compared to other alternative “green” solvents and extraction techniques used by Matić et al. [26].

It is known that in rodents and many other experimental animals, carotenoids are subjected to cleavage by BCO1 and BCO2 immediately after digestion and absorption, and therefore, they are present in the serum at very low levels. This enzymatic activity leads to the formation of vitamin A-active compounds and apocarotenoids. Vitamin A plays a pivotal role throughout the vertebrate life cycle and is known as the vitamin with the broadest spectrum of actions [12,17]. The biological function of vitamin A that stands out the most is its role in transcriptional regulation through retinoic acid, which binds to nuclear retinoic acid receptors and regulates the expression of hundreds of genes [30].

Given that β-carotenoid is found in the highest concentration in applied pumpkin pulp extract, its fate in rats under in vivo conditions is of particular interest. An in vivo study on the conversion of β-carotenoid was conducted on animals with vitamin A-sufficient and vitamin A-deficient diets and subjected to β-caroten substitution. It was established that the oxidative conversion of β-carotene by either central, sequential eccentric, or random cleavage leads mainly to the formation of retinoids (vitamin A group of molecules) and that only 5% of the cleavage products are β-apocarotenoids [17,19]. Based on the above statement, we acknowledge that the effects exerted by the application of pumpkin pulp extract containing carotenoids of a determined type and concentration are most likely due to the metabolites resulting from the enzymatic actions of BCO1 and BCO2.

Many long-term cancer survivors end up dying as a result of heart failure rather than the primary disease [26]. Doxorubicin-induced cardiotoxicity (DIC) is a life-threatening consequence, and therefore, the research of agents, primarily herbal preparations, for the prevention of DIC is of exceptional importance. The use of doxorubicin on animals in this research led to a worsening of the animals’ general condition, characterized by thinned fur, bleeding from the nose and gastrointestinal tract, lethargy, the presence of serohemorrhagic content, and coagulation in the chest, as well as ascites. Similar or the same changes were noted in previous studies [6,31], while there is evidence to suggest that ascites is a consequence of doxorubicin-induced heart failure [32,33]. Data on the effect of doxorubicin on body weight are numerous and contradictory [34,35,36,37]. In our experiment, doxorubicin-treated animals gained significantly less body weight compared to the control group, indicating the harmful effects of doxorubicin on metabolism and growth. In studies of DIC that did not observe differences in BW, it would be helpful and informative to determine whether the mass of organs and the skeletal–muscular system increases, or whether the increase in body weight is a consequence of ascites, interstitial edema, or cardiomyocyte edema recorded in these animals [38]. A higher BW gain in animals receiving pumpkin pulp extract (P and PD groups) indicates that the extract positively affects metabolism and body weight in both healthy and doxorubicin-treated animals. However, it is important to note that there is a lack of direct evidence in the existing literature regarding the impact of pumpkin pulp extracts, or individual carotenoids, on the general health of animals. This highlights the significance of this research as one of the first steps in exploring the biological effects of pumpkin pulp extracts, carotenoids, and/or their derivatives, particularly concerning body weight regulation. A study on adolescents established a positive correlation between serum vitamin A levels and BMI, which could explain and support our results [39].

Regarding heart weight, the data are contradictory. In some studies, the weight of the heart decreased as expected after the administration of doxorubicin [34,36], while in other studies, it was greater compared to the group treated with a cardioprotective substance [37]. Carotenoids serve as a substrate for the synthesis of retinoic acid, the transcriptionally active form of vitamin A, which is involved in regulating cardiac function and growth during embryogenesis, as well as under pathological conditions. Under pathological conditions in adult mice, retinoic acid-mediated signaling has been implicated in cardiac remodeling, including the suppression of cardiac hypertrophic gene features [30]. However, it is essential to note that the weight of the heart is influenced by numerous factors, as well as the individual characteristics of each animal. Additional uncertainty in the interpretation of the data is introduced by the fact that the heart weight at the beginning of the trial cannot be determined, making it difficult to interpret the change in heart weight that occurs during the administration of the substances used.

In response to increased stress in the ventricular wall combined with elevated diastolic and pulmonary capillary pressure, the prohormone NT-proBNP is released from the heart ventricles [40]. The role of elevated NT-proBNP values has long been recognized in the diagnosis of various cardiovascular diseases; however, recent focus has been placed on the elevation of this marker as an early predictor of DIC. The study by Yildirim et al. documented the correlation between the serum level of NT-proBNP and echocardiographic parameters in patients who received doxorubicin and developed DIC after a prolonged period. Since common biomarkers previously extensively studied were not found to be specific for DIC, the study by Yildirim et al. indicates that NT-proBNP could be an effective early marker of subclinical DIC, even when there are no echocardiographic changes [40]. Another study, involving children, also highlights the importance of monitoring NT-proBNP levels to prevent chronic DIC, as statistically significantly elevated levels of this marker were observed after 6 months of therapy [41]. In our research, doxorubicin led to a statistically significant increase in the level of NT-proBNP in groups D and ND compared to the control group.

Contrary to the study by Bansal et al., which stated that the administration of nebivolol, a cardioprotective agent, did not reduce serum NT-proBNP levels [42], our study demonstrated that pumpkin pulp extract containing carotenoids alleviated the toxic effects of doxorubicin and statistically significantly reduced NT-proBNP values compared to group D. Due to the administration of toxic doses of doxorubicin in this study, a statistically significant increase in hsTnT values was noted in groups D and ND, while the values of CK-MB, of the same groups, did not differ significantly compared to the control group. Administration of the extract reduced the serum values of both tested biomarkers; however, this was not statistically significant compared to the D group. There are no other studies involving carotenoids extracted from pumpkin pulp to compare our results, but research has been conducted on the effect of administering carotenoids of different origins. Lycopene has been proven to prevent DIC by reducing oxidative stress and cardiomyocyte damage through the regulation of CK-MB [43]. Carotenoids found in plants from the pumpkin family affect the reduction of doxorubicin-induced elevated CK-MB levels and are effective in the therapy against cardiovascular diseases of other etiologies [44]. These contradictory results suggest that measuring the value of NT-proBNP to diagnose and predict the further course of DIC would be more adequate.

The tissue damage and remodeling as a consequence of doxorubicin-induced cardiotoxicity have been demonstrated in numerous histopathological studies. Changes that are most often recorded and analyzed in previous studies include interstitial and cardiomyocyte edema, myofibril disorganization, inflammatory cell infiltration, vacuolization, and cardiomyocyte disorganization [33,36,45,46,47,48,49]. In addition to the above, the presence of hemorrhagic areas, as well as changes in the appearance of cardiomyocyte nuclei, was also recorded in the heart tissue of the animals in the conducted research. The problem we noticed during the literature search is the inconsistency in the type of changes recorded, the way these changes are evaluated, and the lack of a uniform scoring system. For this reason, during our team’s previous research, we developed the doxorubicin damage score (DDS), which encompasses all the changes mentioned in the research on DIC, along with clear criteria for evaluating them [50]. As expected, the DDS score was negative (implying the absence of myocardial damage) in the control C group, as well as the N and P groups. All tissue alterations recorded in the DDS scoring were abundantly present in the myocardium of the D and ND group animals, leading to significantly higher DDS scores, which correspond to moderate tissue damage. At the cellular level, DIC is initiated by the excessive accumulation of ROS in the mitochondria and an increase in proinflammatory cytokines, all leading to morphological changes constituting the DDS score—the creation of inflammatory infiltrates, the accumulation of neutrophils in the heart tissue, changes in the appearance of the nucleus in terms of the appearance of karyopyknosis and a perinuclear halo, as well as a series of other histopathological changes, which eventually lead to apoptosis or some other type of regulated cell death and even necrosis of the heart tissue [36,51]. The application of pumpkin pulp extract containing carotenoids (group PD) significantly alleviated myocardium tissue damage induced by doxorubicin, and the mentioned histological changes were absent or present to a lesser extent in this group compared to the D and ND groups. One can stipulate that the extract of pumpkin pulp, through carotenoids or their derivatives, prevents or reduces the occurrence of the mentioned histopathological changes precisely by inhibiting apoptosis and preventing excessive accumulation of ROS.

Doxorubicin-induced damage to cardiomyocytes, as documented through H&E-stained tissue slides and the DDS score, was even more evident through the decrease in TnI expression in the D and ND groups. Loss of TnI in a cell indicates a disturbance of the cell membrane, most likely due to increased ROS accumulation and OS, which is a well-documented mechanism of doxorubicin toxicity [35,52,53]. Reestablishing TnI expression after the administration of pumpkin pulp strongly suggests that carotenoids exert antioxidant effects in cardiomyocytes and thus protect cells. Three parameters of expression were analyzed, and it appears that carotenoids primarily affected the extent of staining, i.e., the number of cells expressing TnI, which supports the conclusion that carotenoids protect the integrity of cardiomyocytes and thus prevent TnI loss. These results are supported by previously conducted studies that applied anti-cardiac troponin I antibody [52,53]. Herman et al. also documented a decrease in TnI expression with elevated serum hsTnT values at much lower doses of doxorubicin. Such results indicate the sensitivity of the monitored parameters [52].

Apoptosis is considered a crucial step in the development of DIC, which affects the loss of contractile function in the heart and, consequently, leads to heart failure. This mechanism has been demonstrated in animals and cell culture [44,48,49]. Apoptosis occurs through the mitochondrial pathway, which can be determined by decreased expression of Bcl2 and increased expression of Casp3 [49,54,55]. Members of the Bcl2 family have pro- or antiapoptotic effects. They are significant in the regulation of apoptosis, which places them in focus when it comes to research on drugs that promote the antiapoptotic effect of Bcl2 [56]. Studies have shown that the administration of doxorubicin leads to a decrease in Bcl-2 expression [46,53,57], which is consistent with the results of our study. Proapoptotic effects of doxorubicin were documented through a decrease in the expression of Bcl2 and an increase in the expression of Casp3 in animals treated with doxorubicin (groups D and ND). This doxorubicin-induced effect was partially blocked by co-administration of the extract. The antiapoptotic potential of pumpkin pulp extract may be a direct effect of the Bcl2 family or an indirect effect due to its antioxidative actions. Pumpkin pulp extract containing the mentioned provitamin A carotenoids may exert antioxidative and antiapoptotic effects through vitamin A activity, which is supported by previous research. The antioxidant capacity of vitamin A given to rats (25 IU/kg/day) is the basis of its ability to protect the heart from doxorubicin-induced toxicity [58]. Oxidative stress due to doxorubicin therapy increased the antioxidant activity of endogenous vitamin A [59]. In an animal model of myocardial ischemia/reperfusion injury, the application of 10 mg/kg/day of the active retinoid isoform all-trans retinoic acid attenuated the oxidative stress-induced apoptosis. In this model, it was concluded, however, that antiapoptotic effects were more clearly linked to the upregulation of pro-survival mechanisms by retinoic acid receptors, rather than the direct antioxidant activity of vitamin A [60].

Besides the vitamin A pathway, pleiotropic effects of the extract and its compounds could be achieved by acting on some of the signaling pathways that are at the crossroads of oxidative and apoptotic pathways. We believe that such a pathway could involve Nrf2, as Nrf2 is involved in both apoptotic and oxidative pathways. Studies show that Nrf2-mediated upregulation of the antiapoptotic protein Bcl-2 leads to a decrease in the Bcl-2-associated X-protein gene, the release of cytochrome c from mitochondria, caspase activation, as well as a significant reduction in apoptotic cell death and an increase in cell survival [61].

In physiological conditions, Nrf2 is present in the cytoplasm of cells [62]. A small number of previous studies have performed immunohistochemical analyses of Nrf2 antibodies on heart tissue in chronic DIC, concluding that reduced cytoplasmic Nrf2 is a result of Nrf2 being transferred from the cytoplasm to the nucleus, which is caused by doxorubicin-induced damage [57,63,64]. It is said that in oxidative stress conditions, its dislocation in the nucleus initiates a series of cascade reactions [62]. This explains and supports our findings of decreased Nrf2 expression in animals treated with doxorubicin. There is evidence that antioxidant enzymes such as SOD, CAT, and GPx, as well as ROS accumulation, are associated with Nrf2 status. The authors claim that the absence of Nrf2 in the cytoplasm and disruption of the Nrf2/HO-1 molecular pathway strongly damages mitochondria and leads to excessive ROS production and OS [48,65]. Increased Nrf2 after pumpkin pulp extract treatment implies that this mixture of carotenoids exerts antioxidative properties and affects Nrf2 (and possibly Nrf2/HO1 pathway) either directly as a transcription factor or indirectly by decreasing ROS and OS. Considering that oxidative stress plays a leading role in the development of DIC, it is necessary to find substances that prevent or at least alleviate DIC through the regulation of Nrf2 [64]. Pumpkin pulp extracted in natural solvents, such as NADES, could be one of these substances.

Inflammation and cell proliferation are two vital processes in the tissue of any organ, and doxorubicin affects both. The nuclear protein Ki67 is most often used as a marker of tumor cell proliferation, but it may be affected by other, non-tumorous conditions. Its role as a marker of interphase and mitotic cells is increasingly highlighted, and its distribution changes significantly during cell cycle progression [66]. Recognizing that heart regeneration (involving the creation of new cardiomyocytes) is induced in the presence of cardiomyocyte-damaging agents, the importance of understanding cell cycle activity and identifying cells in a specific phase is highlighted [67]. Doxorubicin application exhibited a proinflammatory profile, increased COX2 expression, and induced Ki67. On the contrary, the application of pumpkin pulp extract effectively neutralized the toxic effects of doxorubicin, and a decrease in the expression of COX2- and Ki67-positive cells was recorded. However, no studies were found in the literature that examined the influence of the mixture or individual carotenoids on the expression of the aforementioned markers, so it is difficult to provide support or refute the obtained results, which opens the possibility for further research. Vitamin A/A/retinoids are products of the oxidative metabolism of β-carotene and are essential for mammalian embryogenesis, including heart formation. In cardiogenesis, retinoic acid exerts effects on the proliferation and fate of multipotent cardiac progenitor cells (reducing proliferation, promoting cardiomyocyte differentiation, and determining their atrial or ventricular fate). New evidence suggests that they may regulate cardiac regeneration, postnatal cardiac function, and the progression of cardiovascular disease [68]. The regeneration of lost myocardium after myocardial infarction (MI) using the injection of stem cell-derived cardiomyocytes into the infarcted heart has been studied as a potential therapeutic strategy. In this setting, retinoic acid has emerged as a general inducer of differentiation of pluripotent stem cells (PSCs) toward cardiomyocytes [69,70]. In vitro studies have shown that all-trans retinoic acid (ATRA) regulates the differentiation of H9c2 cardiomyoblasts, suggesting that it has the potential to be an inducer of differentiation in cardiac progenitor cells (CPCs) [68].

Vitamin A has effects not just on PSCs and CPCs but also on cells of the healthy and diseased postnatal myocardium. Administration of ATRA decreased the proliferation of primary cardiac fibroblasts in vitro [14,71], suggesting its anti-fibrotic effects. In contrast, dietary vitamin A supplementation (0.3 mg/kg, administered daily) in rats suffering from MI prevented hypertrophy [68].

It is worth mentioning that carotenoids are potent molecules: their practical application is often limited due to low absorption. Absorption from lipid-based solvents is 70% or more, and it imposes the need for new solvents that would increase the solubility capacity of carotenoids, enhance the extraction and stabilization of natural products, and also meet ecological standards and be completely nontoxic for nature, animals, and humans [14]. This highlights NADES as an effective carotenoid extraction model, offering a sustainable and nontoxic alternative that aligns with ecological and safety standards. Additionally, a particularly significant aspect of this research is that, despite being a new generation of solvents and primarily investigated as extraction solvents, NADES are not widely investigated in in vivo research. Accordingly, it is essential to highlight that this study also investigates their safety and potential applications.

Administration of NADES solely (N group) did not impair animals’ general condition and body weight. Additionally, the occurrence of ascites, pleural fluid, and coagulum was absent. Its nontoxic nature is confirmed by the absence of significant differences when the N group is compared to the control group regarding cardiovascular markers (CK-MB, NT-proBNP, hsTnI), tissue morphology assessed through DDS score, and expression of TnI, Nrf2, Bcl-2, COX2, and Ki67. Additionally, when comparing the effects of NADES alone to the co-treatment of NADES and doxorubicin, results indicate that NADES did not interfere with doxorubicin’s effects—neither mitigating nor exacerbating its impact. This finding is particularly relevant as it suggests that NADES can be safely used as an extraction medium without introducing confounding physiological effects, reinforcing its potential for future applications in functional food and pharmacological research.

Although there are no precise data in the literature regarding the toxic or protective effects of NADES on the myocardium, some studies have shown that administering it in low doses does not have a toxic effect on the overall health of animals [72,73,74]. These studies provide a basis for further development and application of NADES in the pharmaceutical industry or for medical purposes.

## 5. Conclusions

The examination of cardiotoxicity caused by doxorubicin was conducted through an extensive review on several levels at which doxorubicin manifests its unwanted effects, and the results obtained within this research are in agreement with studies conducted in recent years. Likewise, the NADES (C8:C10) solvent, applied alone or in combination with doxorubicin, did not show adverse effects or potentiate the toxic effects of doxorubicin, indicating that it is safe for animal use. This highlights NADES as an effective carotenoid extraction model, providing a sustainable and nontoxic alternative that aligns with ecological and safety standards.

Finally, the results of this study strongly suggest that the active substances in pumpkin pulp extract, specifically carotenoids and their presumed oxidative derivatives, which are likely to be vitamin A active compounds, have a cardioprotective effect. Considering that it did not show any adverse effects in the aspects examined, we firmly believe that it could be used in the prevention of DIC. In addition, the conducted research is in agreement with the literature data indicating that the mixture of carotenoids or their derivatives exhibits a strong synergistic effect, and therefore more easily prevents damage. Further investigation of this extract will enable the safe and effective integration of herbal preparations into clinical settings, thereby expanding the range of treatment options available to patients and ultimately improving healthcare outcomes. To address the issue of preventing doxorubicin’s side effects, it is necessary to answer many more questions and examine a large number of promising substances. Our study is just a drop in the ocean of these efforts, and during the preparation, the authors themselves noticed several shortcomings that, for the sake of science and the betterment of patients, must not remain unmentioned. Firstly, no single species provides a perfect model for studying all aspects of carotenoids in humans, and the extrapolation of data obtained in rodents to humans must be done with caution. Secondly, the composition of the pumpkin pulp extract was thoroughly investigated in terms of its carotenoid composition and concentration. However, its further metabolism in animals and the presence of other substances, apart from carotenoids, that could contribute to the preventive action of the extract in terms of doxorubicin-induced cardiotoxicity, were not investigated.

## Figures and Tables

**Table 2:** Average number of Casp3-, COX2-, and Ki67-positive cells per visual field (VP) and staining intensity.

	Average Number of Casp3+ per VP	Median Expression Intensity (Casp3+)	Average Number of COX2+ per VP	Median Expression Intensity (COX2+)	Average Number of Ki67+ per VP	Median Expression Intensity (Ki67+)
C	3.38 ± 0.75	1	3.78 ± 1.03	1	1.90 ± 0.75	1
N	3.98 ± 0.54 ^#^	1 ^#^	3.91 ± 0.84 ^#^	1 ^#^	2.23 ± 0.27 ^#^	1 ^#^
P	3.54 ± 0.84 ^#^	1 ^#^	3.84 ± 0.72 ^#^	1 ^#^	2.14 ± 0.42 ^#^	1 ^#^
D	6.81 ± 1.02 *	2 *	5.91 ± 1.11 *	2 *	11.18 ± 1.39 *	2 *
ND	4.90 ± 0.49 *^,#^	1 ^#^	4.33 ± 0.59 ^#^	1	9.70 ± 1.43 *^,#^	2 *
PD	3.84 ± 0.799 ^#^	1 ^#^	4.39 ± 0.49 ^#^	1 ^#^	7.75 ± 0.52 ^#^	2 *

* statistically significant difference compared to group C, *p* < 0.001. ^#^ statistically significant difference compared to group D, *p* < 0.001.

## Data Availability

The original contributions presented in this study are included in the article/Supplementary Materials. Further inquiries can be directed to the corresponding author.

## Supplementary Materials

The following supporting information can be downloaded at: https://www.mdpi.com/article/10.3390/pharmaceutics17080977/s1.

## References

[1] Hammo A.A., Althanoon Z.A., Ahmad A.A. (2022). The Protective Effect of Coenzyme Q10 against Doxorubicin-induced Nephrotoxicity in Albino Rats. Rev. Electrónica Vet..

[2] Wenningmann N., Knapp M., Ande A., Vaidya T.R., Ait-Oudhia S. (2019). Insights into Doxorubicin-induced Cardiotoxicity: Molecular Mechanisms, Preventive Strategies, and Early Monitoring. Mol. Pharmacol..

[3] Wang B., Yu Y., Zhang Y., Hao X., Yang S., Zhao H., Sun Q., Wang Y. (2021). Right ventricular dysfunction in patients with diffuse large B-cell lymphoma undergoing anthracycline-based chemotherapy: A 2D strain and 3D echocardiography study. Int. J. Cardiovasc. Imaging.

[4] Perez M.J., Briz O. (2009). Bile-acid-induced cell injury and protection. World J. Gastroenterol..

[5] Zhu R., Wang Y., Zhang L., Guo Q. (2012). Oxidative stress and liver disease. Hepatol. Res..

[6] Lebrecht D., Setzer B., Rohrbach R., Walker U.A. (2004). Mitochondrial DNA and its respiratory chain products are defective in doxorubicin nephrosis. Nephrol. Dial. Transplant..

[7] Kadaja L., Eimre M., Paju K., Roosimaa M., Poedramaegi T., Kaasik P., Pehme A., Orlova E., Mudist M., Peet N. (2010). Impaired oxidative phosphorylation in overtrained rat myocardium. Exp. Clin. Cardiol..

[8] Wallace K.B., Sardão V.A., Oliveira P.J. (2020). Mitochondrial Determinants of Doxorubicin Induced Cardiomyopathy. Circ. Res..

[9] Okoro C.O., Fatoki T.H. (2023). A Mini Review of Novel Topoisomerase II Inhibitors as Future Anticancer Agents. Int. J. Mol. Sci..

[10] Poliakov E., Uppal S., Rogozin I.B., Gentleman S., Redmond T.M. (2020). Evolutionary aspects and enzymology of metazoan carotenoid cleavage oxygenases. Biochim. Biophys. Acta Mol. Cell Biol. Lipids.

[11] Nagata M., Yamashita I. (1992). Simple method for simultaneous determination of chlorophyll and carotenoids in tomato fruit. Nippon Shokuhin Kogyo Gakkaishi.

[12] Widjaja-Adhi M.A.K., Golczak M. (2020). The molecular aspects of absorption and metabolism of carotenoids and retinoids in vertebrates. Biochim. Biophys. Acta Mol. Cell Biol. Lipids.

[13] Malkanthi A., Hiremath U.S. (2020). Pumpkin powder (*Cucurbita maxima*)-supplemented string hoppers as a functional food. Int. J. Food Nutr. Sci..

[14] Stupar A., Šeregelj V., Ribeiro B.D., Pezo L., Cvetanović A., Mišan A., Marrucho I. (2021). Recovery of β-carotene from pumpkin using switchable natural deep eutectic solvents. Ultrason. Sonochemistry.

[15] Dai Y., Witkamp G.J., Verpoorte R., Choi Y.H. (2015). Tailoring properties of natural deep eutectic solvents with water to facilitate their applications. Food Chem..

[16] Lee C.M., Boileau A.C., Boileau T.W., Williams A.W., Swanson K.S., Heintz K.A., Erdman J.W. (1999). Review of animal models in carotenoid research. J. Nutr..

[17] Martínez A., Cantero J., Meléndez-Martínez A.J., Paulino M. (2022). A Computer Simulation Insight into the Formation of Apocarotenoids: Study of the Carotenoid Oxygenases BCO1 and BCO2 and Their Interaction with Putative Substrates. Molecules.

[18] Bohn T., de Lera A.R., Landrier J.F., Rühl R. (2023). Carotenoid metabolites, their tissue and blood concentrations in humans and further bioactivity via retinoid receptor-mediated signalling. Nutr. Res. Rev..

[19] Barua A.B., Olson J.A. (2000). beta-carotene is converted primarily to retinoids in rats in vivo. J. Nutr..

[20] Bungau S., Abdel-Daim M.M., Tit D.M., Ghanem E., Sato S., Maruyama-Inoue M., Yamane S., Kadonosono K. (2019). Health Benefits of Polyphenols and Carotenoids in Age-Related Eye Diseases. Oxidative Med. Cell. Longev..

[21] Zhuang C., Yuan J., Du Y., Zeng J., Sun Y., Wu Y., Gao X.-H., Chen H.-D. (2022). Effects of Oral Carotenoids on Oxidative Stress: A Systematic Review and Meta-Analysis of Studies in the Recent 20 Years. Front. Nutr..

[22] Frangiamone M., Cimbalo A., Font G., Alonso-Garrido M., Manyes L. (2021). In vitro exposure to pumpkin extract induces a protective transcriptomic profile in blood brain barrier electron transport chain. Rev. Toxicol..

[23] Saha S., Buttari B., Panieri E., Profumo E., Saso L. (2020). An Overview of Nrf2 Signaling Pathway and Its Role in Inflammation. Molecules.

[24] Mansouri A., Reiner Ž., Ruscica M., Tedeschi-Reiner E., Radbakhsh S., Bagheri Ekta M., Sahebkar A. (2022). Antioxidant Effects of Statins by Modulating Nrf2 and Nrf2/HO-1 Signaling in Different Diseases. J. Clin. Med..

[25] Bosanac M., Amidzic J., Stefanovic M., Radic J., Kolarov-Bjelobrk I., Janicic S., Lazic B., Djokanovic D., Misan A., Cvetkovic B. (2023). Can pumpkin save us of doxorubicin induced cardiotoxicity?. Int. J. Morphol..

[26] Matić M., Stupar A., Pezo L., Ilić N.Đ., Mišan A., Teslić N., Pojić M., Mandić A. (2024). Eco-Friendly Extraction: A green approach to maximizing bioactive extraction from pumpkin (*Curcubita moschata* L.). Food Chem. X.

[27] Rošul M., Đerić N., Mišan A., Pojić M., Šimurina O., Halimi C., Nowicki M., Cvetković B., Mandić A., Reboul E. (2022). Bioaccessibility and uptake by Caco-2 cells of carotenoids from cereal-based products enriched with butternut squash (*Cucurbita moschata* L.). Food Chem..

[28] Ezzat S.M., Salem M.A., El Mahdy N.M., Mahfouz M.M., Nabavi S.M., Silva A.S. (2022). Chapter 4.9—Lecithin. Antioxidants Effects in Health.

[29] Adadi P., Barakova N.V., Krivoshapkina E.F. (2018). Selected Methods of Extracting Carotenoids, Characterization, and Health Concerns: A Review. J. Agric. Food Chem..

[30] Holloway C., Zhong G., Kim Y.K., Ye H., Sampath H., Hammerling U., Isoherranen N., Quadro L. (2022). Retinoic acid regulates pyruvate dehydrogenase kinase 4 (Pdk4) to modulate fuel utilization in the adult heart: Insights from wild-type and β-carotene 9′,10′ oxygenase knockout mice. FASEB J..

[31] Patil P.P., Khanal P., Patil V.S., Charla R., Harish D.R., Patil B.M., Roy S. (2022). Effect of *Theobroma cacao* L. on the Efficacy and Toxicity of Doxorubicin in Mice Bearing Ehrlich Ascites Carcinoma. Antioxidants.

[32] Greene R.F., Collins J.M., Jenkins J.F., Speyer J.L., Myers C.E. (1983). Plasma pharmacokinetics of adriamycin and adriamycinol: Implications for the design of in vitro experiments and treatment protocols. Cancer Res..

[33] Najafi M., Hooshangi Shayesteh M.R., Mortezaee K., Farhood B., Haghi-Aminjan H. (2020). The role of melatonin on doxorubicin-induced cardiotoxicity: A systematic review. Life Sci..

[34] El-Refaiy A.I., Salem Z.A., Badawy A.A., Dahran N., Desouky M.A., El-Magd M.A. (2025). Protective effects of lemon and orange peels and olive oil on doxorubicin-induced myocardial damage via inhibition of oxidative stress and inflammation pathways. Front. Pharmacol..

[35] Zaki S.M., Algaleel W.A., Imam R.A., Abdelmoaty M.M. (2019). Mesenchymal stem cells pretreated with platelet-rich plasma modulate doxorubicin-induced cardiotoxicity. Hum. Exp. Toxicol..

[36] Reis-Mendes A., Ferreira M., Padrão A.I., Duarte J.A., Duarte-Araújo M., Remião F., Carvalho F., Sousa E., Bastos M.L., Costa V.M. (2024). The Role of Nrf2 and Inflammation on the Dissimilar Cardiotoxicity of Doxorubicin in Two-Time Points: A Cardio-Oncology In Vivo Study Through Time. Inflammation.

[37] Rajangam J., Krishnan N., Palei N.N., Bhatt S., Das M.K., Das S., Mathusoothanan K. (2022). Ameliorative Potential of Rosuvastatin on Doxorubicin-induced Cardiotoxicity by Modulating Oxidative Damage in Rats. Turk. J. Pharm. Sci..

[38] Postmus A.C., Kruit J.K., Eilers R.E., Havinga R., Koster M.H., Johmura Y., Nakanishi M., van de Sluis B., Jonker J.W. (2023). The chemotherapeutic drug doxorubicin does not exacerbate p16^Ink4a^-positive senescent cell accumulation and cardiometabolic disease development in young adult female LDLR-deficient mice. Toxicol. Appl. Pharmacol..

[39] Su X., Patel N., Zhu S., Zhou X., Chen Y., Chen J., Mo X. (2024). Association between serum vitamin A and body mass index in adolescents from NHANES 1999 to 2006. Sci. Rep..

[40] Yildirim A., Tunaoglu F.S., Kambur K., Pinarli F.G. (2013). The utility of NT-proBNP and various echocardiographic methods in the determination of doxorubicin induced subclinical late cardiotoxicity. Pol. Heart J..

[41] El Amrousy D., El-Afify D., Khedr R., Ibrahim A.M. (2022). Omega 3 fatty acids can reduce early doxorubicin-induced cardiotoxicity in children with acute lymphoblastic leukemia. Pediatr. Blood Cancer.

[42] Bansal N., Adams M.J., Ganatra S., Colan S.D., Aggarwal S., Steiner R., Amdani S., Lipshultz E.R., Lipshultz S.E. (2019). Strategies to prevent anthracycline-induced cardiotoxicity in cancer survivors. Cardiooncology.

[43] Liu C., Ma X., Zhuang J., Liu L., Sun C. (2020). Cardiotoxicity of doxorubicin-based cancer treatment: What is the protective cognition that phytochemicals provide us?. Pharmacol. Res..

[44] Mukherjee P.K., Singha S., Kar A., Chanda J., Banerjee S., Dasgupta B., Haldar R.K., Sharma N. (2022). Therapeutic importance of Cucurbitaceae: A medicinally important family. J. Ethnopharmacol..

[45] Zhang Y.Y., Yi M., Huang Y.P. (2017). Oxymatrine Ameliorates Doxorubicin-Induced Cardiotoxicity in Rats. Cell. Physiol. Biochem..

[46] Ekinci Akdemir F.N., Yildirim S., Kandemir F.M., Tanyeli A., Küçükler S., Bahaeddin Dortbudak M. (2021). Protective effects of gallic acid on doxorubicin-induced cardiotoxicity; an experimantal study. Arch. Physiol. Biochem..

[47] Alharbi F.K., Alshehri Z.S., Alshehri F.F., Alhajlah S., Khalifa H.A., Dahran N., Ghonimi W. (2023). The role of hesperidin as a cardioprotective strategy against doxorubicin-induced cardiotoxicity: The antioxidant, anti-inflammatory, antiapoptotic, and cytoprotective potentials. Open Vet. J..

[48] Ahmed A.Z., Mumbrekar K.D., Satyam S.M., Shetty P., D’Souza M.R., Singh V.K. (2021). Chia Seed Oil Ameliorates Doxorubicin-Induced Cardiotoxicity in Female Wistar Rats: An Electrocardiographic, Biochemical and Histopathological Approach. Cardiovasc. Toxicol..

[49] Dundar H.A., Kiray M., Kir M., Kolatan E., Bagriyanik A., Altun Z., Aktas S., Ellidokuz H., Yilmaz O., Mutafoglu K. (2016). Protective Effect of Acetyl-L-Carnitine Against Doxorubicin-induced Cardiotoxicity in Wistar Albino Rats. Arch. Med. Res..

[50] Alhazzani K., Alotaibi M.R., Alotaibi F.N., Aljerian K., As Sobeai H.M., Alhoshani A.R., Alanazi A.Z., Alanazi W.A., Alswayyed M. (2021). Protective effect of valsartan against doxorubicin-induced cardiotoxicity: Histopathology and metabolomics in vivo study. J. Biochem. Mol. Toxicol..

[51] Zare M.F.R., Rakhshan K., Aboutaleb N., Nikbakht F., Naderi N., Bakhshesh M., Azizi Y. (2019). Apigenin attenuates doxorubicin induced cardiotoxicity via reducing oxidative stress and apoptosis in male rats. Life Sci..

[52] Herman E.H., Lipshultz S.E., Rifai N., Zhang J., Papoian T., Yu Z.X., Takeda K., Ferrans V.J. (1998). Use of cardiac troponin T levels as an indicator of doxorubicin-induced cardiotoxicity. Cancer Res..

[53] Herman E.H., Zhang J., Lipshultz S.E., Rifai N., Chadwick D., Takeda K., Yu Z.-X., Ferrans V.J. (1999). Correlation between serum levels of cardiac troponin-T and the severity of the chronic cardiomyopathy induced by doxorubicin. J. Clin. Oncol..

[54] Al-Amir H., Janabi A., Hadi N.R. (2023). Ameliorative effect of nebivolol in doxorubicininduced cardiotoxicity. J. Med. Life.

[55] Karatas O., Balci Yuce H., Tulu F., Taskan M.M., Gevrek F., Toker H. (2020). Evaluation of apoptosis and hypoxia-related factors in gingival tissues of smoker and non-smoker periodontitis patients. J. Periodontal Res..

[56] Qian S., Wei Z., Yang W., Huang J., Yang Y., Wang J. (2022). The role of BCL-2 family proteins in regulating apoptosis and cancer therapy. Front. Oncol..

[57] Li D., Liu X., Pi W., Zhang Y., Yu L., Xu C., Sun Z., Jiang J. (2022). Fisetin Attenuates Doxorubicin Induced Cardiomyopathy In Vivo and In Vitro by Inhibiting Ferroptosis Through SIRT1/Nrf2 Signaling Pathway Activation. Front. Pharmacol..

[58] Tesoriere L., Ciaccio M., Valenza M., Bongiorno A., Maresi E., Albiero R., Livrea M.A. (1994). Effect of vitamin A administration on resistance of rat heart against doxorubicin-induced cardiotoxicity and lethality. J. Pharmacol. Exp. Ther..

[59] Danelisen I., Palace V., Lou H., Singal P.K. (2002). Maintenance of myocardial levels of vitamin A in heart failure due to adriamycin. J. Mol. Cell. Cardiol..

[60] Zhu Z., Zhu J., Zhao X., Yang K., Lu L., Zhang F., Shen W., Zhang R. (2015). All-Trans Retinoic Acid Ameliorates Myocardial Ischemia/Reperfusion Injury by Reducing Cardiomyocyte Apoptosis. PLoS ONE.

[61] Niture S.K., Jaiswal A.K. (2012). Nrf2 protein up-regulates antiapoptotic protein Bcl-2 and prevents cellular apoptosis. J. Biol. Chem..

[62] Zhao X., Tian Z., Sun M., Dong D. (2023). Nrf2: A dark horse in doxorubicin-induced cardiotoxicity. Cell Death Discov..

[63] Bai Y., Chen Q., Sun Y., Wang X., Lv L., Zhang L., Liu J., Zhao S., Wang X. (2017). Sulforaphane protection against the development of doxorubicin-induced chronic heart failure is associated with Nrf2 Upregulation. Cardiovasc. Ther..

[64] Qi W., Boliang W., Xiaoxi T., Guoqiang F., Jianbo X., Gang W. (2020). Cardamonin protects against doxorubicin-induced cardiotoxicity in mice by restraining oxidative stress and inflammation associated with Nrf2 signaling. Biomed. Pharmacother..

[65] Chen Q.M. (2022). Nrf2 for protection against oxidant generation and mitochondrial damage in cardiac injury. Free Radic. Biol. Med..

[66] Sun X., Kaufman P.D. (2018). Ki-67: More than a proliferation marker. Chromosoma.

[67] Liu X., Pu W., He L., Li Y., Zhao H., Li Y., Liu K., Huang X., Weng W., Wang Q.-D. (2021). Cell proliferation fate mapping reveals regional cardiomyocyte cell-cycle activity in subendocardial muscle of left ventricle. Nat. Commun..

[68] Liang X., Chen M., Wang D., Wen J., Chen J. (2021). Vitamin A deficiency indicating as low expression of LRAT may be a novel biomarker of primary hypertension. Clin. Exp. Hypertens..

[69] Szewczyk K., Chojnacka A., Górnicka M. (2021). Tocopherols and Tocotrienols-Bioactive Dietary Compounds; What Is Certain, What Is Doubt?. Int. J. Mol. Sci..

[70] Wolfensohn S., Lloyd M. (1994). Handbook of Laboratory Animal Management and Welfare.

[71] Christidi E., Brunham L.R. (2021). Regulated cell death pathways in doxorubicin-induced cardiotoxicity. Cell Death Dis..

[72] Mišan A., Nađpal J., Stupar A., Pojić M., Mandić A., Verpoorte R., Choi Y.H. (2020). The perspectives of natural deep eutectic solvents in agri-food sector. Crit. Rev. Food Sci. Nutr..

[73] Paiva A., Craveiro R., Aroso I., Martins M., Reis R.L., Duarte A.R.C. (2014). Natural deep eutectic solvents–solvents for the 21st century. ACS Sustain. Chem. Eng..

[74] Liu Y., Friesen J.B., McAlpine J.B., Lankin D.C., Chen S.N., Pauli G.F. (2018). Natural Deep Eutectic Solvents: Properties, Applications, and Perspectives. J. Nat. Prod..

