# Supplementary figures and images for: Potential of Pumpkin Pulp Carotenoid Extract in the Prevention of Doxorubicin-Induced Cardiotoxicity
Source: Pharmaceutics. 2025 Jul 28;17(8):977. doi: 10.3390/pharmaceutics17080977 (PMC12389520; doi:10.3390/pharmaceutics17080977)

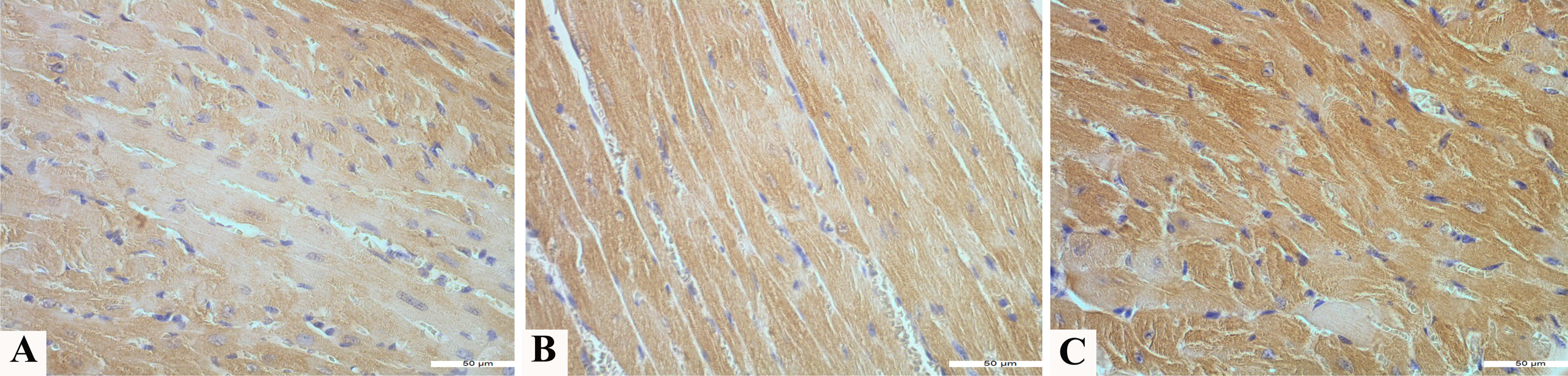

Supplement: Supplementary file 1 [file pharmaceutics-17-00977-s001.zip › pharmaceutics-3760615-supplementary/ALL FIGURES/Sup1.jpg]

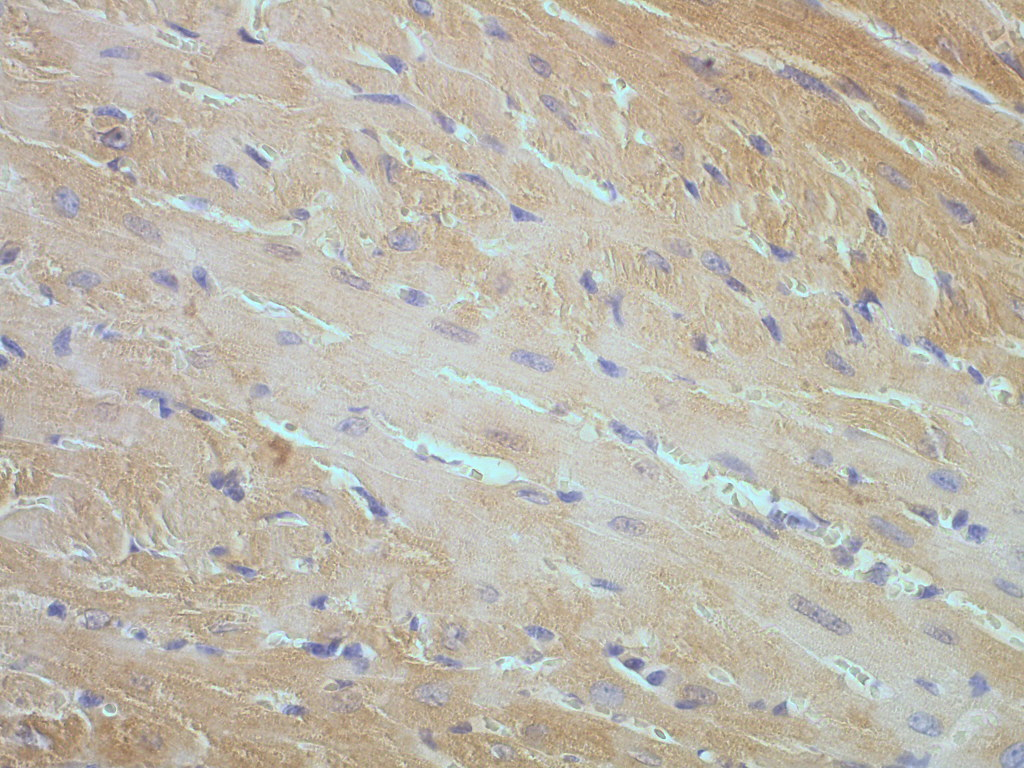

Supplement: Supplementary file 1 [file pharmaceutics-17-00977-s001.zip › pharmaceutics-3760615-supplementary/ALL FIGURES/Sup1A.tif]

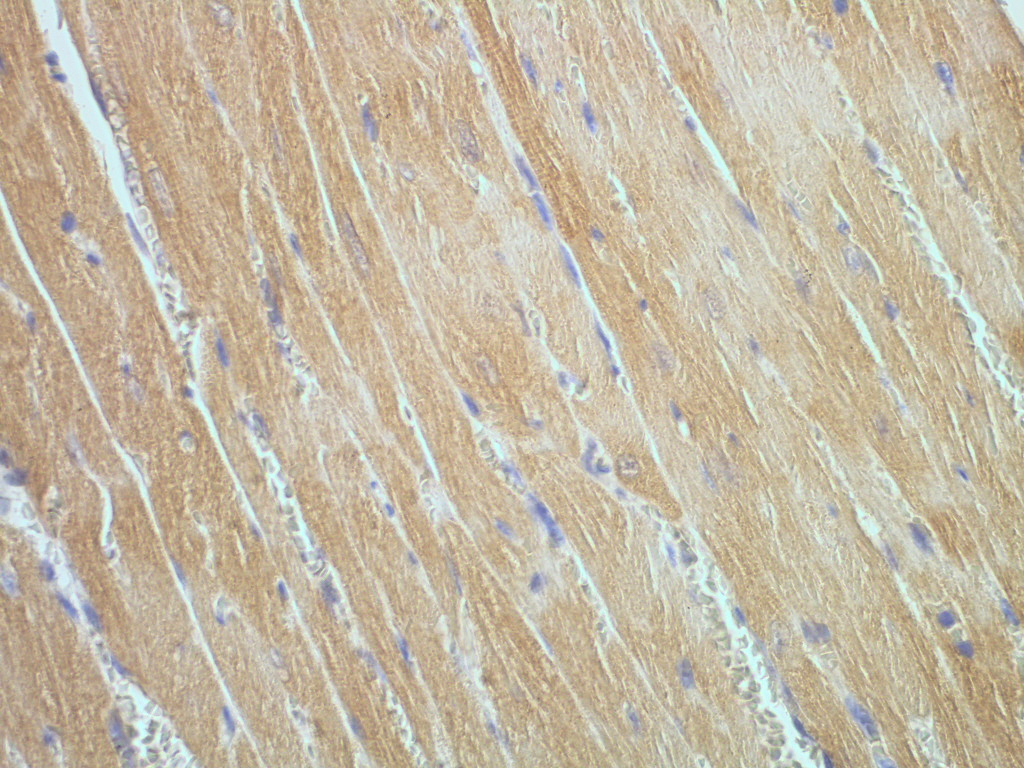

Supplement: Supplementary file 1 [file pharmaceutics-17-00977-s001.zip › pharmaceutics-3760615-supplementary/ALL FIGURES/Sup1B.tif]

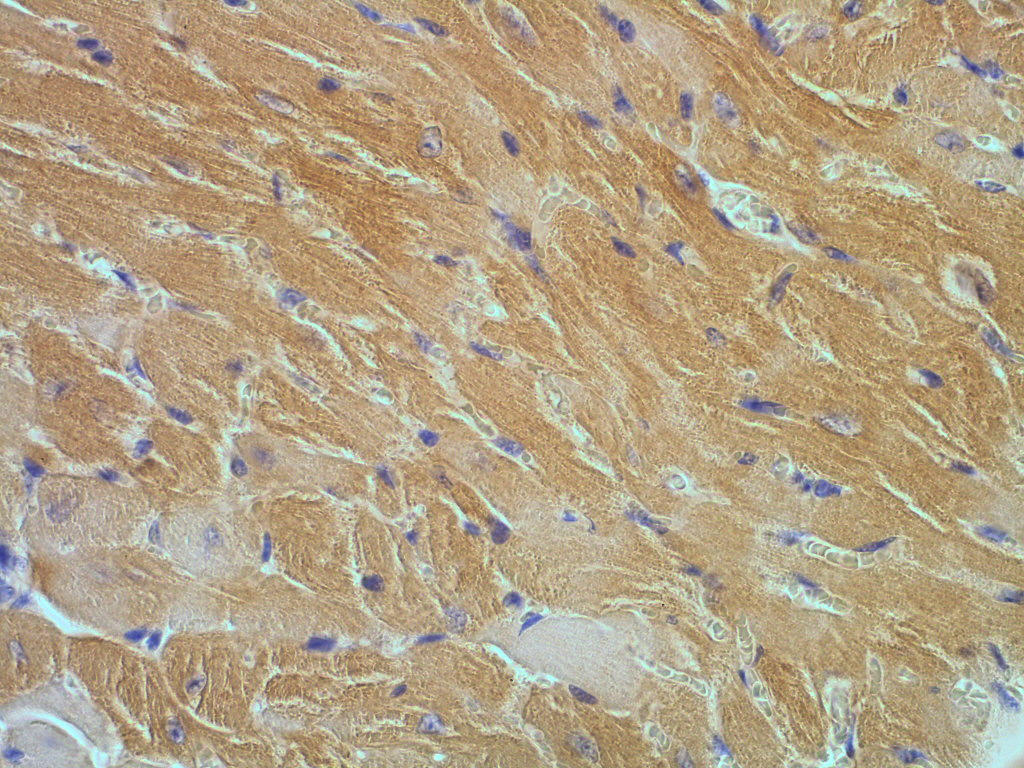

Supplement: Supplementary file 1 [file pharmaceutics-17-00977-s001.zip › pharmaceutics-3760615-supplementary/ALL FIGURES/Sup1C.tif]

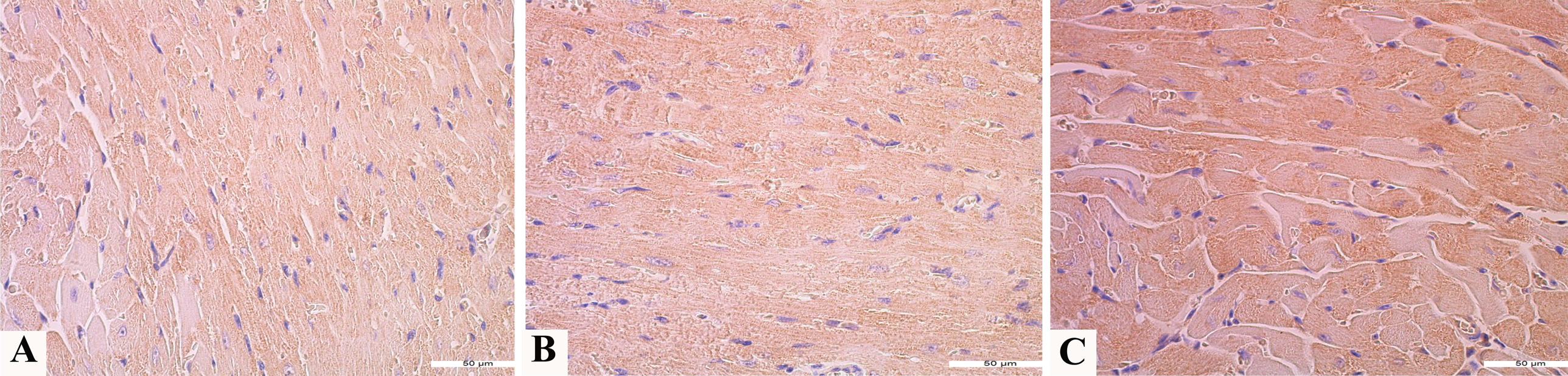

Supplement: Supplementary file 1 [file pharmaceutics-17-00977-s001.zip › pharmaceutics-3760615-supplementary/ALL FIGURES/Sup2.jpg]

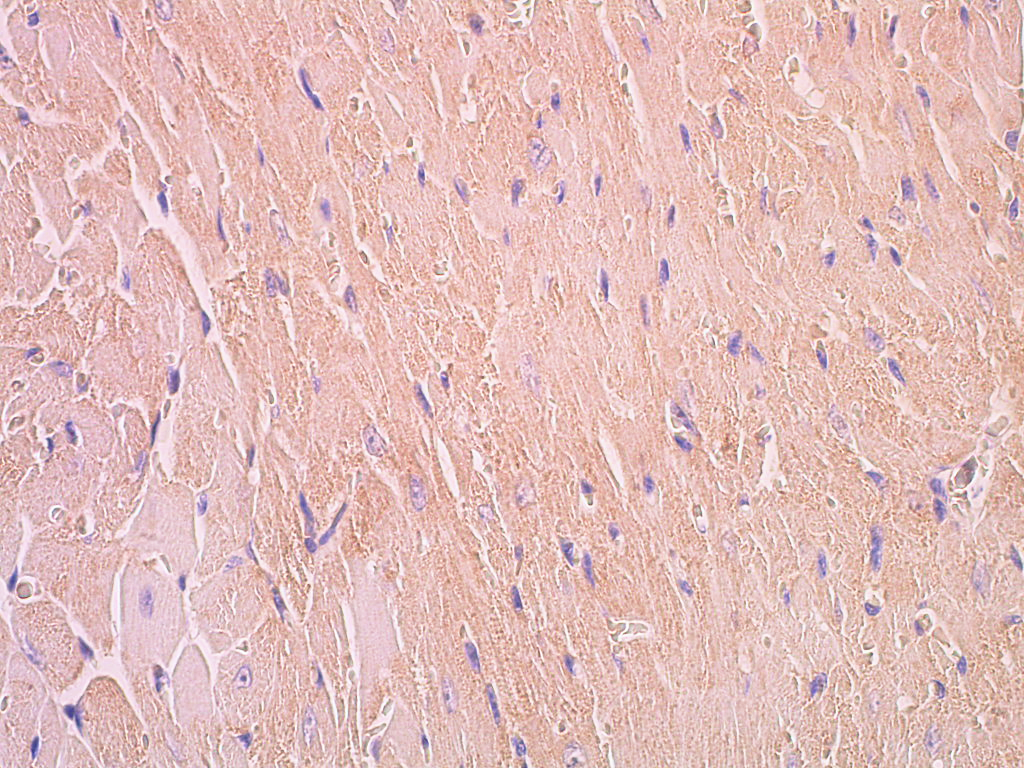

Supplement: Supplementary file 1 [file pharmaceutics-17-00977-s001.zip › pharmaceutics-3760615-supplementary/ALL FIGURES/Sup2A.tif]

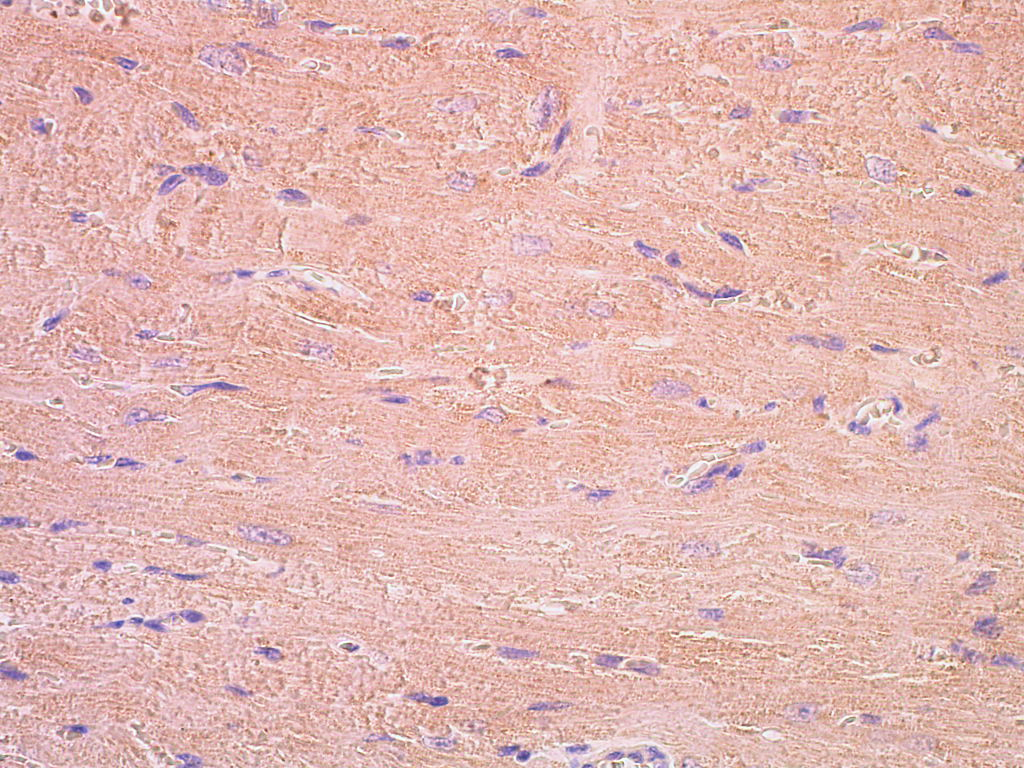

Supplement: Supplementary file 1 [file pharmaceutics-17-00977-s001.zip › pharmaceutics-3760615-supplementary/ALL FIGURES/Sup2B.tif]

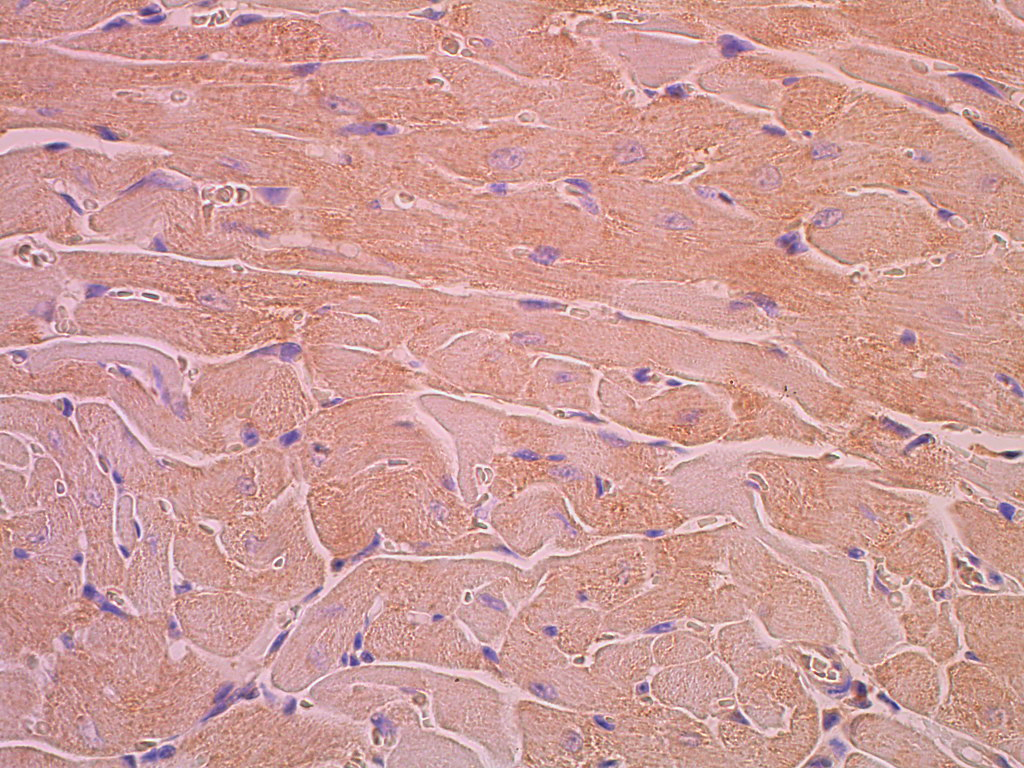

Supplement: Supplementary file 1 [file pharmaceutics-17-00977-s001.zip › pharmaceutics-3760615-supplementary/ALL FIGURES/Sup2C.tif]

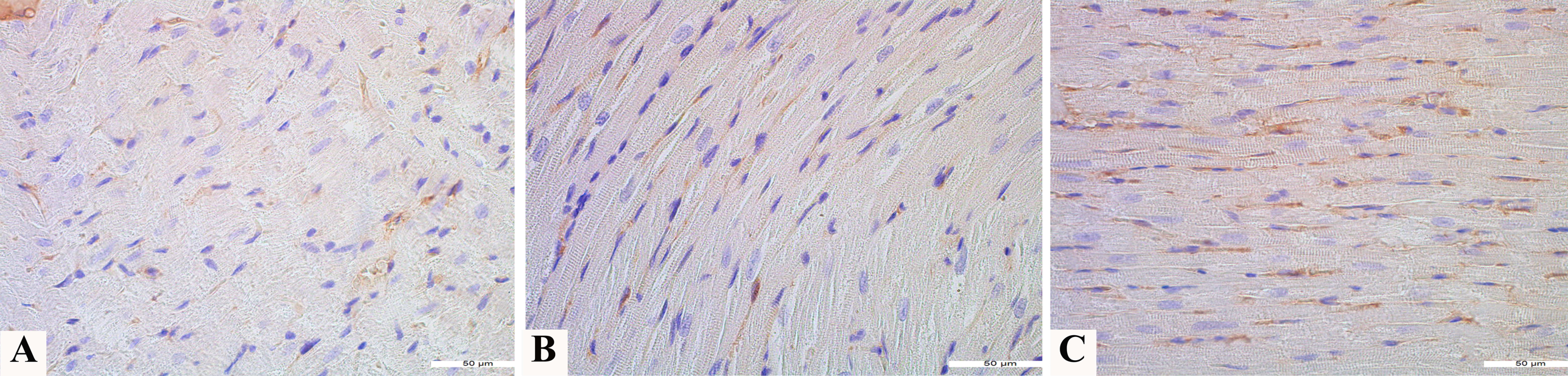

Supplement: Supplementary file 1 [file pharmaceutics-17-00977-s001.zip › pharmaceutics-3760615-supplementary/ALL FIGURES/Sup3.jpg]

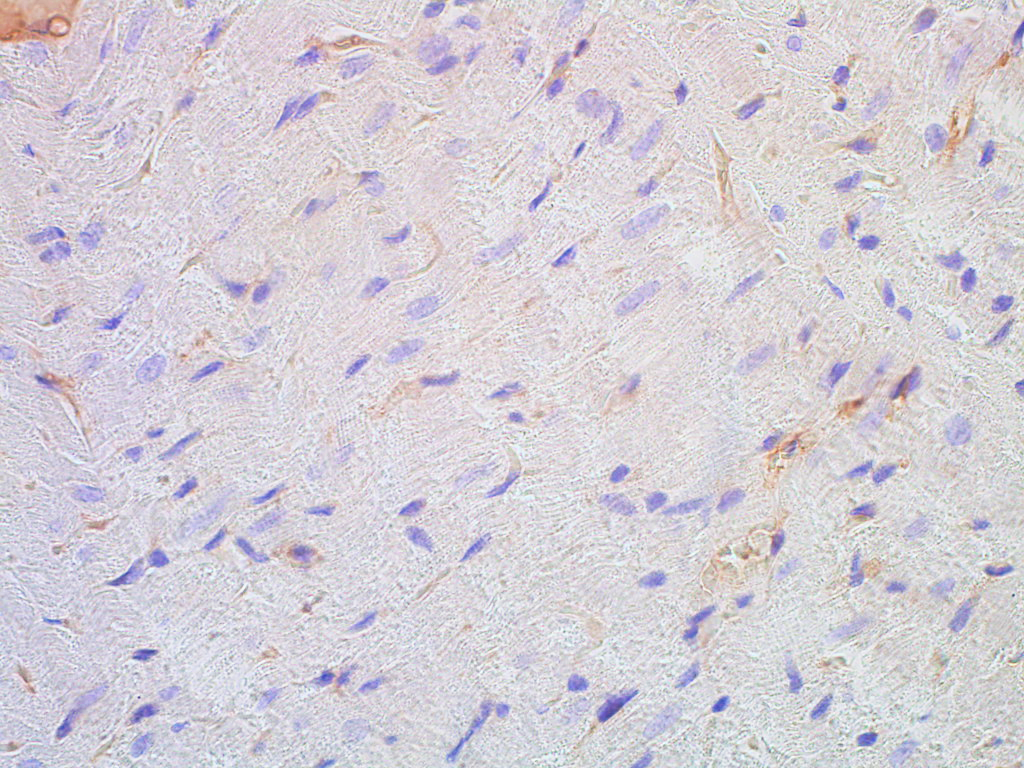

Supplement: Supplementary file 1 [file pharmaceutics-17-00977-s001.zip › pharmaceutics-3760615-supplementary/ALL FIGURES/Sup3A.tif]

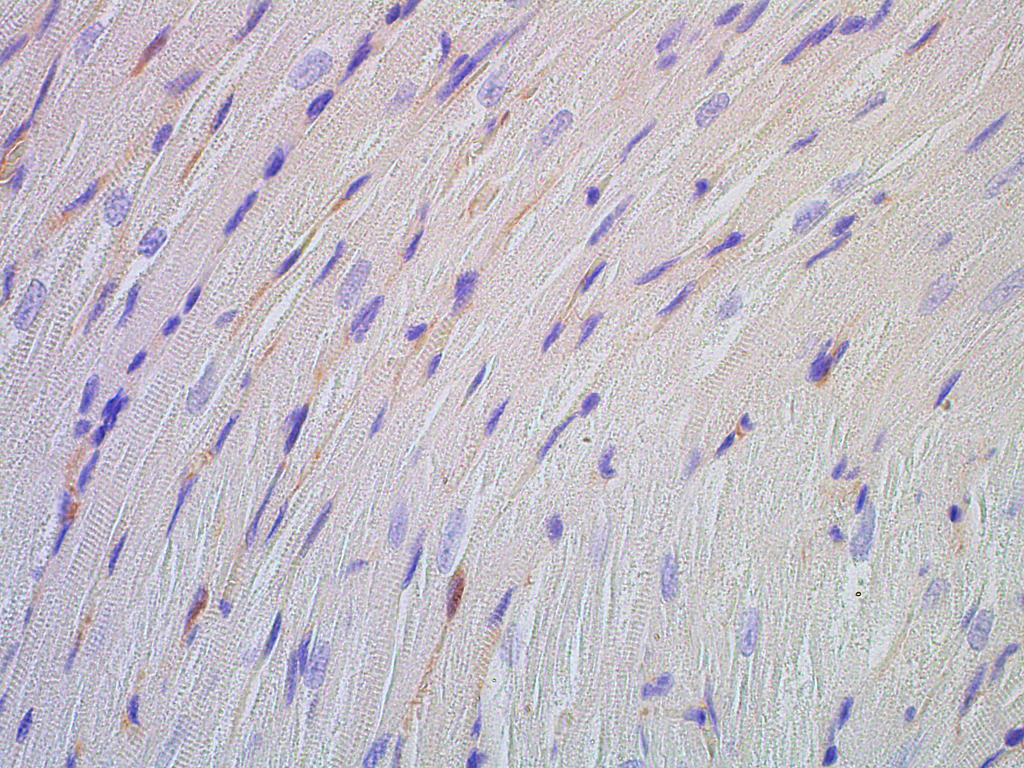

Supplement: Supplementary file 1 [file pharmaceutics-17-00977-s001.zip › pharmaceutics-3760615-supplementary/ALL FIGURES/Sup3B.tif]

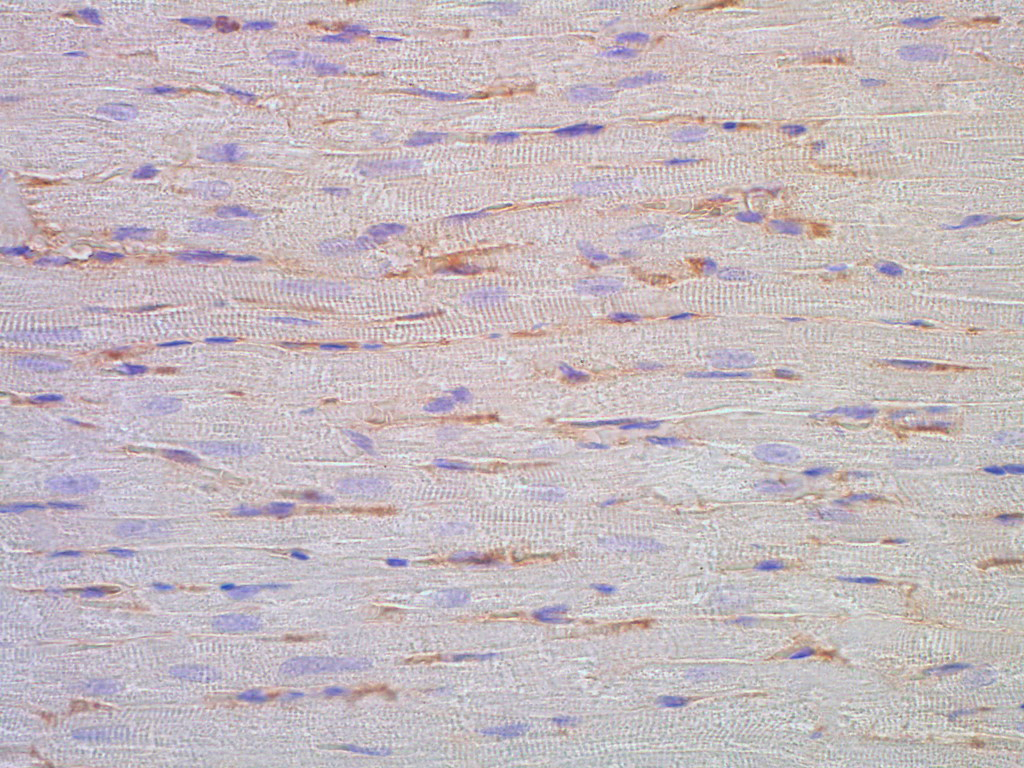

Supplement: Supplementary file 1 [file pharmaceutics-17-00977-s001.zip › pharmaceutics-3760615-supplementary/ALL FIGURES/Sup3C.tif]
